# Hybridization in closely related *Rhododendron* species: half of all species-differentiating markers experience serious transmission ratio distortion

**DOI:** 10.1002/ece3.1570

**Published:** 2015-07-07

**Authors:** Tobias Marczewski, David F Chamberlain, Richard I Milne

**Affiliations:** 1 Royal Botanic Garden Edinburgh20A Inverleith Row, Edinburgh, EH3 5LR, UK; 2 Institute of Molecular Plant Sciences, University of EdinburghMayfield Road, Edinburgh, EH9 3JH, UK

**Keywords:** Hybridization, *Rhododendron*, species differentiation, AFLP

## Abstract

An increasing number of studies of hybridization in recent years have revealed that complete reproductive isolation between species is frequently not finalized in more or less closely related organisms. Most of these species do, however, seem to retain their phenotypical characteristics despite the implication of gene flow, highlighting the remaining gap in our knowledge of how much of an organism’s genome is permeable to gene flow, and which factors promote or prevent hybridization. We used AFLP markers to investigate the genetic composition of three populations involving two interfertile *Rhododendron* species: two sympatric populations, of which only one contained hybrids, and a further hybrid-dominated population. No fixed differences between the species were found, and only 5.8% of the markers showed some degree of species differentiation. Additionally, 45.5% of highly species-differentiating markers experienced significant transmission distortion in the hybrids, which was most pronounced in F1 hybrids, suggesting that factors conveying incompatibilities are still segregating within the species. Furthermore, the two hybrid populations showed stark contrasting composition of hybrids; one was an asymmetrically backcrossing hybrid swarm, while in the other, backcrosses were absent, thus preventing gene flow.

## Introduction

### Speciation as a continuum

While many biologists still favor the biological species concept, which relies on complete reproductive isolation to delineate species, several authors highlight the problems that this approach might have with regard to understanding the underlying processes that actually drive speciation (Abbott et al. 2008; Mallet 2008; Abbott et al. 2013). Complete isolation is merely the end-point of the divergence process, but effective barriers to gene flow due to factors like ecology, geography, and phenology can arise well before full isolation is achieved (Butlin et al. 2008; Lexer and Widmer 2008; Nosil and Feder 2012). Moreover, some entities retain genetic and morphological distinctness even in the face of ongoing gene flow, due to strong selection on parts of the genome (Wu 2001; Via 2009). Therefore, an increasing number of evolutionary biologists are adopting a genic view of species that emphasizes the role of genes and genetic architecture (how alleles of several genes interact to cause a phenotype) in the process of adaptation, hereby relaxing the constraint of complete reproductive isolation of species. This shift in focus is of importance, as speciation is an ever ongoing process, and complete reproductive isolation is just one stage in the diversification process, albeit a very important one (Hewitt 2001). An understanding of the genomic basis involved in adaptation in combination with identifying factors leading to reproductive isolation will allow questions related to biodiversity to be addressed in a more unifying manner.

To understand how species might achieve complete reproductive isolation in the absence of a geographic barrier, we need to understand how much gene flow nascent species can tolerate while maintaining the potential to diverge, and how this gene flow might contribute to the evolutionary future of species (Abbott et al. 2013). Nascent or recently diverged species pairs may exist almost anywhere along a continuum from complete interbreeding to complete reproductive isolation. Processes responsible for divergence, and the maintenance of species barriers, may vary depending on both the stage of divergence (Nosil and Feder 2012) and the taxa involved (Lexer and Widmer 2008). Moreover, variation in hybrid zone structure for a particular species pair between localities indicates a likely role for ecology in these processes at a local level (e.g., Milne et al. 2003). Therefore, full understanding of these processes will require examination of species pairs at different stages of divergence and encompassing a wide taxonomic and geographic range (Hewitt 2001). Hybrid zones span the near entirety of this continuum, as they can, for example, be formed in early stages between populations that differ hardly at all (Barton and Hewitt 1989; Via 2009), or may represent later stages, for example, when species enter a phase of contact after having diverged for a certain time in allopatry (Barton and Hewitt 1985; Abbott et al. 2013). Genomic compatibility is generally related to time since divergence (Presgraves 2010). Furthermore, signals in the genetic architecture due to differential adaptation are likely to become more difficult to identify the further the groups have diverged, as unrelated genomic incompatibilities accumulate (Via 2009). Although initial adaptation begins the process of divergence, speciation can only occur if genomic incompatibilities accumulate to prevent merging of the nascent species, for example, in the face of changing environmental conditions. Hence not only is it important to investigate the genetic basis for adaptation, but it is also necessary to understand how, in light of gene flow, species retain group-specific gene pools along the speciation continuum. Therefore, large species complexes which are still in early stages of divergence, and in which members have not yet evolved complete reproductive isolation, are especially promising for detecting genes involved in incompatibilities and hence divergence.

### Study species

**Figure 1:**
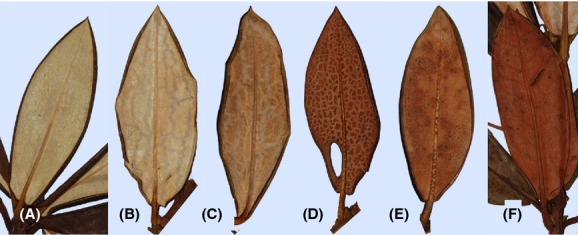
Morphological types of lower leaf surface hair cover (indumentum) in *Rhododendron aganniphum*, *R. phaeochrysum*, and intermediates. (A) *R. aganniphum*: long hairs forming a thick, densely felted indumentum, white on mature leaves; (F) *R. phaeochrysum*: indumentum comprising short hairs, always turning red-brown on older leaves; (B, C) forms close to A, but indumentum splitting during maturation of leaves, and sometimes turning cream-brown; (D) hairs markedly shorter than in A, indumentum splitting early in development, turning patchy and red-brown in mature leaves; (E) close to F, but patches of agglutinated slightly longer hairs visible on mature leaves.

The uplift of the Himalayas, the last ∼4 Ma of which have been rather rapid (Takada and Matsu’ura 2007), caused dramatic climatic (e.g., Donghuai 2004; Sun et al. 2008) as well as geological changes to the region. This caused repeated range fragmentation, and genesis of new habitats, with detectable effects on species composition in some areas (Wu et al. 2006). Moreover, it has triggered substantial radiation events in some plant genera (Liu et al. 2006; Wang et al. 2009), including *Rhododendron* subgenus Hymenanthes, most of whose 200+ species occur in the Himalayas and SW China (Chamberlain 1982; Milne et al. 2010). While its monophyly is well-supported (Kron 1997; Kurashige et al. 2001; Goetsch et al. 2005; Milne et al. 2010), attempts to resolve relationships among Sino-Himalayan members of subgenus Hymenanthes have not met with much success (Milne et al. 2010). This partly reflects a lack of genetic differentiation between species (Milne et al. 2010), but moreover even the most basal species within the subgenus are highly interfertile with one another (Milne, et al. [Bibr b28],[Bibr b29]), and hybrids commonly occur wherever species ranges overlap (Milne, et al. [Bibr b28],[Bibr b29]; Zha, et al. [Bibr b49],[Bibr b50]). Hence, the potential for gene flow and reticulate evolution is high, and this appears to be reflected by incongruence between phylogenies (Goetsch et al. 2005; Eckert and Carstens 2008; Milne et al. 2010). Two species with extensively overlapping ranges and potential for gene flow are *Rhododendron aganniphum* and *R. phaeochrysum*. These are part of a particularly complex group of species within subsection Taliensia of subgenus Hymenanthes (Chamberlain 1982), within which numerous hybrids are known or suspected. Both *R. aganniphum* and *R. phaeochrysum* occur exclusively in Yunnan and Sichuan, SW China, with respective altitude ranges of 3350–4550 m and 3350–4300 m. Hence, there is considerable overlap between them, but where they are sympatric, *R. phaeochrysum* always dominates the lower altitudes, while *R. aganniphum* is predominantly found at higher altitudes. Above 4000 m, *R. aganniphum* has the habit of an appressed shrub and is covered completely by snow during winter (T. Marczewski pers. obs.). At lower altitudes, it commonly grows to 1.5 m (T. Marczewski pers. obs.), and sometimes 3 m (Chamberlain 1982), indicating that growth form might be determined by ecological constraints. Conversely, *R. phaeochrysum* grows to between 1.2 and 4.5 m, and can be a tree or large shrub but never an appressed shrub. Both species have very similar flowers (white with varying numbers of crimson markings), leaf shape and bark; the easiest character to identify hybrids is the hair structure on the lower leaf surface (Fig. 1). Due to their remote and relatively inaccessible ranges, little is known about the population structures of these species. However, we observed that some areas of sympatry contain large hybrid swarms, whereas other seemingly ecological equivalent settings contain none. Furthermore, a population of putative hybrids was discovered, their morphology matching a hybrid type found in the swarm populations (type E, Fig. 1). In this population, all individuals were similar to one another in morphology, and there was no apparent intergradation with either parent, meaning it was not a typical hybrid swarm. This provided an opportunity to compare sympatric populations with and without hybrids, and two apparently different types of hybrid zone. The questions we addressed were as follows: (1) Is the genetic composition of the two morphologically distinct hybrid zones different? (2) How much of the genome is permeable in a setting with a hybrid swarm? (3) Do markers differentiating the species show different introgression patterns from other loci, and hence are they mostly neutrally accumulated differences or are they affected by incompatibilities? (4) Is there a population-specific component to hybridization, resulting in some alleles present only in one population of a species showing deviations from expected neutral introgression? (5) Is there evidence for past or ongoing introgression in a setting with no hybrids, indicating that hybrids were existent or overlooked?

To address these questions, we examined three sites: a population of apparently segregating hybrids that was sympatric with both parents, a population of morphologically uniform hybrids that grew close to but not overlapping parental ranges, and a site of sympatry where no hybrids were detectable.

## Materials and Methods

### Plant material

Samples of *R. aganniphum*, *R. phaeochrysum*, and putative hybrids were collected in 2007 and 2008 in Yunnan, SW China. The two species are very similar in leaf shape and flower characters, but can be distinguished by leaf indumentum characters. Material was hence assigned to species as follows: In *R. aganniphum*, the indumentum comprises long, ramiform, white hairs which are densely compacted; in *R. phaeochrysum*, the hairs are much shorter, and while cream-white in young leaves, they quickly turn dark brown-red when the leaves are older (Fig. 1). Material with intermediate or mixed indumentum was treated as a putative hybrid.

Two hybrid populations were sampled from Baima Shan mountain, Yunnan. At Site One (28^∘^ 20’ 05.8"N, 99^∘^ 04’ 43.8"E, 4300 m), hybrids exhibited diverse morphology and were sympatric with both parents. From this site were gathered 33 putative hybrids (hyb-1), 37 *R. aganniphum* (aga-1), and 18 *R. phaeochrysum* (pha-1) accessions. Site Two was 6.4 km from Site One (28^∘^ 17’ 25.7"N, 99^∘^ 07’ 15.0"E, 4300 m) and comprised a morphologically relatively uniform population of hybrids with only very few dispersed *R. aganniphum* individuals present; from here, 38 putative hybrids (hyb-2) were collected. Site Three was a different mountain massif, Daxue Shan (28^∘^ 34’ 37.7"N, 99^∘^ 49’ 35.3"E, 4300 m), where the parent species grew sympatrically, but no hybrids were observed. From here, 33 *R. aganniphum* (aga-3) and 25 *R. phaeochrysum* (pha-3) accessions were collected.

Leaves were collected from plants with a minimum spacing of about 5 m to avoid sampling clones and reduce the chance of sampling offspring of the same maternal parent. Collected leaves were transferred to plastic ziplock bags containing silica gel and stored for later DNA extraction.

### DNA extraction and AFLP marker generation

DNA was extracted from silica-dried leaf material with a DNEasy kit (Qiagen, West Sussex, UK), according to the protocol provided by the manufacturer, but using 1.5× the suggested amounts of buffers. DNA content of the extracts was assessed photometrically (NanoVue; GE Healthcare, Buckinghamshire, UK), and a minimum of 50 ng of total DNA was used for each digestion–ligation reaction. For generation of AFLP profiles, the method published by Vos et al. (1995) was followed. The two restriction enzymes EcoRI and MseI were used to cut genomic DNA, and three selective primer pair combinations were used: (1) D2 E-ATC/M-CAG, (2) D3 E-ACT/M-CTA, and (3) D4 E-ATC/M-CGA. EcoRI adapters of the final reactions were labeled with different WellRED dyes (Sigma-Aldrich, Dorset, UK; D2, D3, D4), which are also represented in the first part of each locus name. The pooled fragments were then run on a CEQ 8000 (Beckman Coulter, High Wycombe, UK) and manually scored with CEQ Analysis Software v8.0.

### Error estimation and putative clones

To assess error due to different reactions and scoring, all steps for the generation of the AFLP profiles, apart from the DNA extraction, were duplicated for 14 individuals. For a further two individuals (one from each species), all steps were carried out five times, so that these two individuals were present in each sequencer run performed to generate the AFLP profiles for all individuals.

The error was then calculated as the mean mismatch for all possible pairwise comparisons between AFLP profiles of the same individual. Where two different individuals had a mismatch score no larger than the mean error for the above within-individual comparisons, those two individuals were flagged as being putative clones of one another.

### Marker selection

A total of 344 loci were scored. Afterward, loci for which no population showed allele frequency differences to any other population were removed as uninformative; the function prop.test() in R (R Development Core Team 2010) was used to test whether any two populations had a significantly different dominant allele count. In total, 192 uninformative loci were removed for this reason. Of the 152 retained loci, a further 6 were removed because of missing data, leaving 146 informative loci. Four individuals (two from aga-1, one each from hyb-1 and hyb-2) were also removed from the analysis because of missing data to ensure that all analyzed individuals were scored for all informative markers, leaving 180 analyzed individuals.

### Data analysis: pairwise *F*_ST_

Population allele frequencies were estimated using the Bayesian method from Zhivotovsky (1999). Following note 4 (Zhivotovsky 1999, p. 912), the hyperparameters 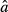 and 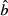 for the beta distribution prior were calculated from the between-locus values of null allele frequencies of the dataset.

From these allele frequency estimates, pairwise population *F*_ST_ values were calculated using the following formula (Hartl and Clark 1997, pp. 123–126): 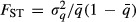, with 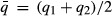 and 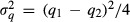, where *q*_1_ and *q*_2_ are the frequencies of the null allele in population 1 and population 2, respectively.

### Data analysis: locus categories

**Figure 2:**
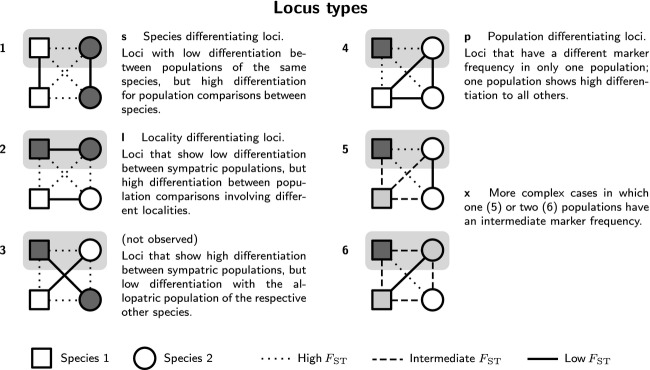
Locus types assigned to loci depending on *F*_ST_ differentiation patterns. The light-gray box indicates that hybrids occur between two sympatric populations of different species at one locality.

In a wider sense, a species is also a group of populations that shares a significant part of its gene pool. Hence, to look at differences between species, one has to identify loci for which the species are differentiated and distinguish them from loci for which only one population shows exceptional differentiation. None of the loci in our data showed fixed differences between the species, likely indicating that none of the markers coincided with a locus directly involved in adaptation or species barriers. On the other hand, some loci showed significant differentiation, suggesting that they either were in close enough proximity in the genome to experience the effects of such loci or that the species have diverged enough to accumulate neutral differences. As there were no fixed differences, but only differences in allele frequency, we decided to call such loci species or population differentiating, instead of species or population specific. To assess whether loci that were species-differentiating behaved differently to other loci with regard to transmission into hybrid offspring, loci were assigned to categories depending on their differentiation patterns inferred from pairwise *F*_ST_ values (Fig. 2).

Loci that showed higher than average differentiation were assigned, based on pairwise *F*_ST_ values, to one or more of the following categories: species differentiating (type s), locality differentiating (type l), population differentiating (type p), or complex (type x).

#### Type s loci

Species-differentiating loci were those that differentiated both populations belonging to the same species from those of the other. Pairwise population *F*_ST_ values for the four parental populations (aga-1, aga-3, pha-1, and pha-3) were grouped into (a) comparisons between populations of the same species and (b) comparisons between populations of different species. Loci were categorized as type s if they satisfied both of the following conditions: (1) The locus showed higher differentiation between species than average observed differentiation within species, meaning that all between-species *F*_ST_ values had to be above the overall mean of all within-species *F*_ST_ values (mean over all loci) and (2) all between-species *F*_ST_ values being at least twice as high as the highest within-species comparison for the locus in question.

If the mean between-species *F*_ST_ fell above the 90% quantile, the locus was additionally classed as highly species differentiating (subtype s^h^).

#### Type l loci

Locality-differentiating loci were those that differentiated the two populations at one locality from those at the other. All pairwise *F*_ST_ values were divided into (a) within-locality (i.e., within Baima Shan or within Daxue Shan) and (b) between-locality comparisons, irrespective of species identity. Loci were then categorized as type l if (1) the locus showed above mean differentiation, meaning in this case that all between-locality *F*_ST_ values had to be above the overall mean of all within-species *F*_ST_ values (mean over all loci), and (2) *F*_ST_ values for all within-locality comparisons were lower than the smallest between-locality comparison.

#### Type p loci

Population-differentiating loci were those that differentiated one population from the other three. To detect these, *F*_ST_ values were first normalized by dividing all comparisons by the highest observed *F*_ST_ value for the locus. Then, for each population, the mean of all pairwise comparisons not involving the population in question was subtracted from the mean of all comparisons involving that population. A score of 1 would indicate a marker frequency unique to the selected population that showed no differences between the other three, whereas 0 or below would indicate that this population was no more different from the others (or less if negative) than they were from each other. However, a marker that strongly differentiated the selected population but also showed differentiation among the other three would also score less than 1. Based on this, loci were categorized as population differentiating for a given population if their score for that population was above 0.67.

#### Type x loci

Additional cases were detected where two populations were strongly differentiated from one another, but one or both of the remaining populations were intermediate, leading to no population having an individual score above 0.67. Such markers were categorized as complex (type x; Fig. 2); see supplementary text for a full explanation of qualification for this category.

### Data analysis: population clustering

For Bayesian clustering, the software Structure (Pritchard et al. 2000) was used. Default parameter settings were used, allowing for admixed individuals, and using the recessive alleles setting (Falush et al. 2007). After checking several runs visually for stationarity of MCMC chains, the burn-in for all runs was set to 20,000 followed by 30,000 iterations. The most likely number of clusters was determined by performing 20 runs for each setting of *K* between one and ten, and then evaluating plots of Δ*K*, as suggested by Evanno et al. (2005). This analysis was performed with the whole dataset and with a dataset containing only individuals from Baima Shan (aga-1, pha-1, hyb-1, and hyb-2). No major differences were detected between the results for these two datasets.

### Data analysis: detection of marker clines

The R package introgress (Gompert and Buerkle 2009, 2010) was used to calculate hybrid indices, estimate marker clines in the hybrid populations, and to compare these to genomewide expectations. To examine the potential role of demography (parapatry of hyb-2 to the parents) on cline patterns (Gompert et al. 2012), two analyses were conducted. The first included hybrid populations hyb-1 and hyb-2 combined, and the second the hybrid population hyb-1 alone, as this was the population sympatric with the parental populations (aga-1, pha-1) that were used to estimate parental allele frequencies. Significance for the deviation from neutral expectations was calculated using the parametric method with 10,000 replications.

### Data analysis: simulation of hybrid populations

Because the clustering results from STRUCTURE turned out unexpected (the hybrids formed a cluster instead of showing admixture), datasets containing inbred early- to later-generation hybrids (F1–F4) were simulated to investigate the resulting clustering in STRUCTURE. Additionally, backcross populations were simulated for comparison and to combine the simulated hybrid populations for analysis in introgress, to check for deviation of marker clines.

For the simulations, allele frequency estimates from the populations aga-1 and pha-1 were used to obtain two parental populations, aga-A and pha-B, comprising 50 individuals each. We decided to use simulated parental populations representing the observed allele frequencies instead of the originally sampled individuals, as for each individual at each locus that has the dominant allele, the second allele was not known. Furthermore, the simulated parental populations would certainly conform to the assumptions (1) that the loci are independent and (2) that the population is in Hardy–Weinberg equilibrium.

Genotypes for parental individuals were generated by assigning two alleles to each locus, where the probabilities of obtaining the dominant or the recessive allele were set to the estimated population frequency of the respective allele. Hence, both alleles present at each locus were known for each individual. The two parental populations simulated in this way were used for all hybrid simulations.

F1 hybrid individuals were generated by randomly drawing one individual from each parental population, and then genotypes for loci were obtained by choosing randomly one allele from each parent at each locus. A hybrid population was generated by repeating this process, whereby alleles were drawn with replacement, so that individuals could contribute differently to the hybrid population.

To generate an inbred F2 hybrid population, an F1 population was created as described above, and then F2s were generated by selfing this F1 population. For later-generation hybrids, this process was repeated, with the resulting hybrid population being selfed.

In the case of backcross (BC) populations, a generated F1 or BC population was used as one parent, while the initial parental population (aga-A or pha-B) served as the other.

Ten populations each of BC1A, BC2A, F1, F2, F3, F4, BC1B, and BC2B hybrids, all comprising 50 individuals, were generated. All were simulated independently, so that no analyzed F1 population was the parent of an F2 population and so on. However, for all simulations, the same parental populations (aga-A, pha-B) were used as the starting point. The size of all populations, including the intermediate-precursor populations used to generate later-generation hybrids, was set to 50 individuals.

#### Structure

Each of the 80 simulated hybrid populations was combined with the parental populations (aga-A, aga-B), and for each resulting dataset, 16 runs for every *K* between one and six were performed, using the settings for the original data.

#### Introgress

For the analysis in introgress, the hybrid populations were combined into ten datasets, so that each dataset contained one simulated hybrid population from each of the eight hybrid classes, thereby mimicking a hybrid swarm. The analysis was carried out using the same setting as for the original data. As the original data suggested that no backcrosses to *R. phaeochrysum* are present in any of the hybrid populations, the cline estimation was additionally carried out with all ten datasets, but the backcrosses to pha-B (BC1B, BC2B) removed.

## Results

### Error estimation and putative clones

The combined error introduced due to different reaction conditions and the scoring process, as estimated by the mismatch between separate AFLP profiles for the same individual, was 2.6% (SD ± 0.97), based on the 146 loci included in further analyses. Three pairs of hybrid individuals had pairwise mismatch scores ≤2.6%, indicating putative clones or shared parentage. All three putative clone pairs were individuals from the hybrid population hyb-2, and in each case, one individual from each pair was removed from the data, resulting in 177 individuals remaining in the analyzed dataset.

### Bayesian clustering with Structure

#### Original data

**Figure 3:**
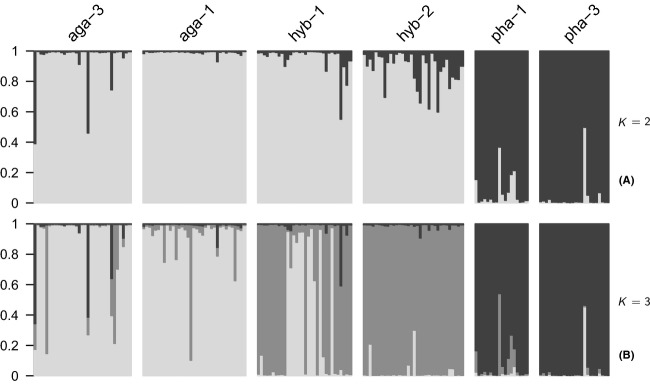
Admixture proportions (*Q*) of individuals in all populations, as estimated by the software Structure. Clearly, the largest value for Δ*K* was observed for *K* = 3. (A) When *K* = 2, populations of the same species cluster together, *Rhododendron aganniphum* (aga-1, aga-3 – light-gray) and *R. phaeochrysum* (pha-1, pha-3 – dark-gray), with both hybrid populations (hyb-1, hyb-2) clustering with *R. aganniphum*, and only few hybrid individuals showing admixture. (B) When *K* = 3, *R. aganniphum* and *R. phaeochrysum* also form separate clusters (light-gray and dark-gray). Instead of showing admixture, the hybrids form their own cluster (medium-gray), to which all individuals of hyb-2 and half of hyb-1 are assigned. The remaining individuals of hyb-1 cluster with *R. aganniphum*.

Plots of Δ*K* (Evanno et al. 2005) unambiguously identified the most likely number of clusters as three. Two of these clusters corresponded to the parental species *R. aganniphum* (aga-1, aga-3) and *R. phaeochrysum* (pha-1, pha-3); however 11 individuals from hyb-1 also clustered with *R. aganniphum*. In addition, a few individuals in the population aga-3 and one individual in pha-3 showed introgression, as indicated by admixture with the respective other species (Fig. 3B). The third cluster comprised all individuals from hyb-2 and the remaining 21 individuals in hyb-1. Moreover, only two individuals from hyb-2 showed any admixture with another cluster, in each case *R. aganniphum*. Hence, the results do not conform to the expectation that the hybrid populations would show an admixture of the two parental populations, assigning most of the hybrids to a third, separate cluster. In comparison, looking at the less likely case of *K* = 2, hybrids showed very low levels of admixture. Individuals of both hybrid populations were in this case mostly assigned to the *R. aganniphum* cluster, with about half the individuals in hyb-2, and only five individuals of hyb-1 showing admixture with the *R. phaeochrysum* cluster (Fig. 3A).

#### Simulated hybrids

**Figure 4:**
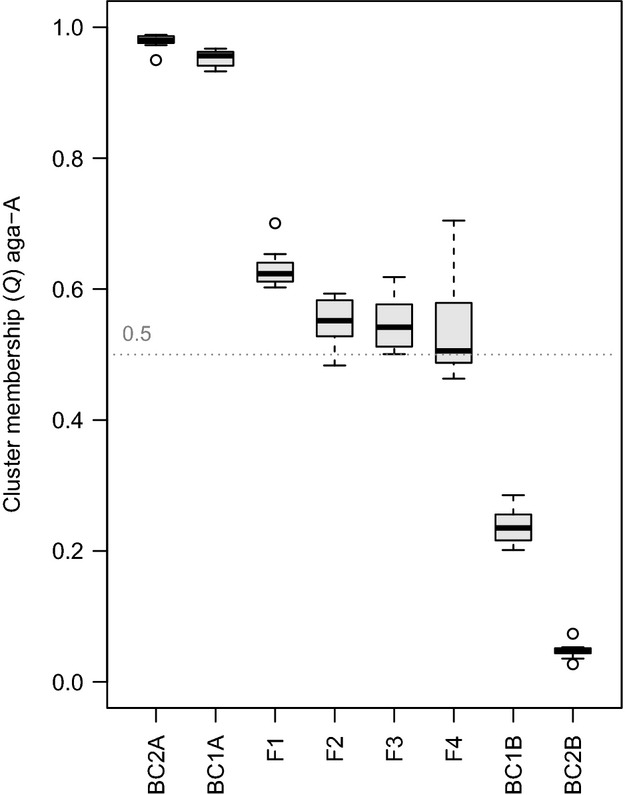
Mean estimates of *Q* for eight simulated types of hybrid population. Ten different populations, each comprising 50 individuals, were simulated for each of the hybrid classes (x-axis). Mean values of *Q* for the populations were calculated based on 16 Structure runs (*K* = 2) performed for each simulated population. Shown is the mean estimated proportion of admixture (*Q*) originating from the cluster assigned to the simulated parental population aga-A.

For all datasets of simulated hybrid populations by far, the highest Δ*K* was observed for *K* = 2. The mean estimates (and ranges of population means) of *Q* for admixture from the *R. aganniphum* cluster (*K* = 2) were as follows: F1 0.63 (0.60–0.70); backcrosses to aga-A – BC1A 0.95 (0.93–0.97), BC2A 0.98 (0.95–0.99); backcrosses to pha-B – BC1B 0.24 (0.20–0.29), BC2B 0.05 (0.03–0.07); F2 0.55 (0.48–0.59); F3 0.55 (0.50–0.62); F4 0.53 (0.46–0.70) (Fig. 4).

**Figure 5:**
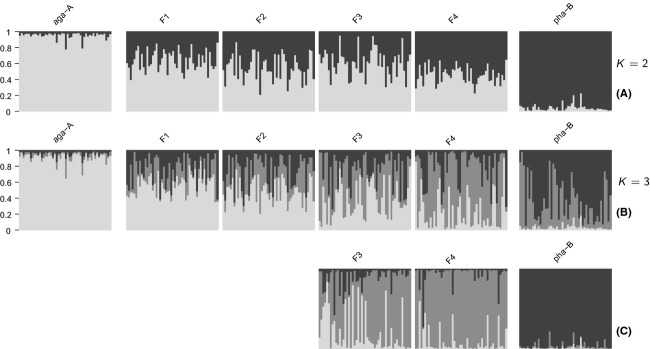
Admixture proportions (*Q*) of individuals of representative simulated later-generation hybrid populations (F1 to F4), as estimated by the software Structure. Parental populations (aga-A, pha-B) are based on allele frequency estimates from the populations aga-1 and pha-1, respectively (see text for details). For all simulated datasets, clearly the highest Δ*K* was *K* = 2. (A) When *K* = 2, all simulated hybrid populations show admixture from both parental clusters (aga-A – light-gray, pha-B – dark-gray). While average estimates of *Q* were equal for both parental clusters in all simulated F2, F3, and F4 populations, average estimates of *Q* in all 10 simulated F1 populations were significantly biased to the aga-A cluster. (B) When *K* = 3, the third cluster (medium-gray) was in most runs attributed to the pha-B population, with later-generation cross-hybrid populations (e.g., F3, F4) showing an increasing contribution from this cluster. (C) In some cases of F3 and F4 populations, this led to the third cluster being exclusively attributed to the hybrid population.

When *K* = 2, all individuals of aga-A were assigned to one cluster, and all individuals from pha-B to the respective other cluster, with a few individuals in both populations, showing low levels of admixture (Fig. 5A). The individuals in all simulated hybrid populations showed clear admixture from both of the parental clusters. While F1s showed on average a significantly higher contribution from the aga-A cluster, this pattern seemed to disappear in later-generation hybrid populations, with average admixture tending toward an equal contribution from both parents. When *K* = 3, individuals of aga-A were still always assigned to one cluster, while individuals of pha-B now showed varying levels of admixture of the remaining two clusters (Fig. 5B). Proceeding from early-generation hybrid populations (F1, F2) to later-generation hybrid populations (F3, F4), an increasing number of individuals got assigned to the third cluster. In several replicates, this led to most hybrid individuals being assigned to the third cluster, while pha-B ceased showing admixture (Fig. 5C).

### Assignment of loci into categories using pairwise *F*_ST_ values

Of the 146 informative loci, 20 loci were species-differentiating (type s), including 11 that were highly so (type s^h^). Only two loci were location-differentiating (type l; loci 21, 22; Table1). A total of 24 loci were population-differentiating, for populations as follows: aga-1, six; aga-3, seven; pha-1, three; pha-3, eight (Table2). Twenty-three loci were complex in their differentiation patterns (type x); these cases ranged from one population being more differentiated from all others, but with intermediate differentiation patterns between the other populations (locus 53, Table1) to sometimes high differentiation between certain population pairs, but no clear pattern (locus 57). Additionally, four type s^h^ markers also satisfied the conditions for type x; this was in all four cases due to a difference in allele frequencies between the two *R. aganniphum* populations (loci 2, 3, 5, 6; Table1). The remaining 77 loci did not show high enough differentiation patterns to fall into any of these categories. Including loci not classed as informative; therefore, 69 of 344 scored markers (20%) were assigned to at least one category (Table3).

**Table 1:** Pairwise population *F*_ST_ values for 146 loci, ordered according to the categories mentioned in Figure 2, and locus-specific significance of transmission distortion in the hybrid population hyb-1 (*P*-value); *P*-values marked with ^*^ are significant for the analysis when including hyb-2. Highly species-differentiating loci are marked with^h^, and loci marked with^x^ additionally fall into category x. *Rhododendron aganniphum* - aga-1 (a1), aga-3 (a3); *R. phaeochrysum* - pha-1 (p1), pha-3 (p3). For type p loci, Pop denotes the population which is differentiated from the others at the respective locus

	Locus	Type	Pop	*F*_ST_ within species		*F*_ST_ between species				*P*-value
				a1¦ a3	p1¦ p3	a1¦ p1	a3¦ p3	a1¦ p3	a3¦ p1	
1	D2_144	s		0.000	0.000	0.246	0.239	0.252	0.233	0.008^*^
2	D2_229	s^h,x^		0.033	0.000	0.177	0.341	0.183	0.334	ns
3	D2_277	s^h,x^		0.052	0.001	0.210	0.450	0.234	0.421	ns
4	D2_285	s^h^		0.000	0.002	0.177	0.217	0.208	0.186	ns
5	D2_294	s^h,x^		0.049	0.003	0.582	0.325	0.554	0.351	0.000^*^
6	D2_302	s^h,x^		0.110	0.010	0.310	0.598	0.256	0.661	ns
7	D4_128	s^h^		0.002	0.002	0.277	0.278	0.235	0.323	ns
8	D4_179	s^h^		0.014	0.010	0.470	0.295	0.411	0.351	0.000^*^
9	D4_238	s^h^		0.007	0.000	0.211	0.280	0.217	0.274	0.001^*^
10	D4_335	s^h^		0.000	0.000	0.474	0.450	0.460	0.465	ns
11	D4_405	s^h^		0.002	0.000	0.310	0.280	0.316	0.274	0.000^*^
12	D2_104	s		0.001	0.010	0.098	0.062	0.051	0.111	ns
13	D2_228	s		0.001	0.003	0.065	0.035	0.048	0.052	ns
14	D3_086	s		0.000	0.000	0.082	0.095	0.094	0.084	ns
15	D3_157	s		0.012	0.003	0.114	0.081	0.147	0.055	ns
16	D3_254	s		0.002	0.002	0.146	0.152	0.119	0.181	ns
17	D4_120	s		0.016	0.000	0.065	0.132	0.070	0.126	0.028^*^
18	D4_121	s		0.001	0.019	0.120	0.062	0.051	0.135	ns
19	D4_125	s		0.000	0.003	0.039	0.059	0.059	0.039	ns
20	D4_178	s		0.000	0.005	0.055	0.084	0.085	0.054	ns
21	D3_065	l		0.077	0.070	0.000	0.001	0.061	0.086	ns
22	D4_112	l		0.215	0.356	0.071	0.021	0.132	0.460	0.000^*^
23	D2_077	p	aga-3	0.283	0.018	0.013	0.297	0.000	0.196	ns
24	D2_138	p	aga-1	0.055	0.009	0.038	0.002	0.070	0.003	ns
25	D2_143	p	pha-3	0.000	0.153	0.000	0.160	0.152	0.000	ns
26	D2_275	p	pha-1	0.000	0.102	0.068	0.002	0.004	0.076	ns
27	D2_307	p	aga-1	0.042	0.001	0.039	0.000	0.048	0.000	ns
28	D2_313	p	aga-3	0.051	0.000	0.001	0.047	0.000	0.043	ns
29	D2_359	p	aga-3	0.043	0.000	0.001	0.044	0.000	0.052	0.000^*^
30	D2_378	p	aga-1	0.046	0.000	0.039	0.000	0.043	0.001	ns
31	D3_076	p	pha-3	0.004	0.148	0.016	0.188	0.234	0.004	ns
32	D3_119	p	aga-1	0.082	0.000	0.074	0.000	0.079	0.001	0.002^*^
33	D3_164	p	pha-3	0.021	0.181	0.008	0.221	0.119	0.003	ns
34	D3_193	p	aga-3	0.079	0.010	0.001	0.036	0.016	0.071	ns
35	D3_198	p	pha-3	0.001	0.191	0.002	0.133	0.156	0.006	ns
36	D3_289	p	aga-3	0.035	0.000	0.001	0.047	0.002	0.043	0.000
37	D3_303	p	pha-3	0.010	0.229	0.056	0.351	0.425	0.021	ns
38	D3_335	p	aga-3	0.092	0.001	0.000	0.108	0.001	0.089	ns
39	D4_067	p	pha-1	0.009	0.124	0.151	0.018	0.002	0.228	ns
40	D4_110	p	aga-1	0.097	0.009	0.144	0.030	0.186	0.008	ns^*^
41	D4_126	p	pha-1	0.000	0.036	0.039	0.000	0.000	0.039	ns
42	D4_147	p	pha-3	0.004	0.218	0.071	0.394	0.420	0.053	ns
43	D4_160	p	aga-1	0.454	0.003	0.520	0.003	0.492	0.012	0.000^*^
44	D4_367	p	pha-3	0.010	0.060	0.011	0.062	0.099	0.000	ns
45	D4_386	p	pha-3	0.000	0.189	0.025	0.274	0.275	0.024	ns
46	D4_391	p	aga-3	0.423	0.000	0.005	0.466	0.007	0.460	ns
Mean (146 loci)				0.035	0.028	0.064	0.082	0.085	0.070	
47	D2_092	x		0.027	0.140	0.098	0.267	0.402	0.026	ns
48	D2_102	x		0.048	0.022	0.126	0.075	0.201	0.024	ns
49	D2_139	x		0.048	0.000	0.024	0.119	0.028	0.114	0.000^*^
50	D2_199	x		0.016	0.062	0.043	0.006	0.003	0.089	ns
51	D2_215	x		0.099	0.054	0.016	0.048	0.011	0.183	ns
52	D3_085	x		0.077	0.019	0.031	0.099	0.002	0.167	ns
53	D3_114	x		0.016	0.198	0.073	0.329	0.425	0.025	ns
54	D3_172	x		0.003	0.060	0.110	0.025	0.013	0.129	ns
55	D3_197	x		0.036	0.003	0.104	0.038	0.142	0.019	ns
56	D3_208	x		0.030	0.062	0.070	0.036	0.000	0.151	ns
57	D3_212	x		0.502	0.022	0.138	0.271	0.055	0.150	ns
58	D3_226	x		0.161	0.003	0.065	0.331	0.041	0.380	ns
59	D3_259	x		0.000	0.060	0.059	0.219	0.224	0.056	ns
60	D3_302	x		0.001	0.034	0.047	0.160	0.147	0.057	ns
61	D3_350	x		0.167	0.018	0.390	0.131	0.492	0.065	ns
62	D4_093	x		0.033	0.019	0.110	0.083	0.208	0.024	0.006^*^
63	D4_129	x		0.058	0.019	0.195	0.016	0.124	0.061	ns
64	D4_168	x		0.027	0.035	0.039	0.049	0.125	0.001	ns
65	D4_198	x		0.042	0.063	0.047	0.029	0.001	0.165	ns
66	D4_202	x		0.000	0.049	0.025	0.113	0.113	0.024	ns
67	D4_260	x		0.126	0.036	0.197	0.075	0.316	0.011	ns
68	D4_296	x		0.000	0.032	0.020	0.101	0.098	0.022	ns
69	D4_313	x		0.038	0.022	0.049	0.030	0.110	0.001	0.047^*^
70	D2_066			0.016	0.002	0.003	0.014	0.000	0.005	ns
71	D2_071			0.004	0.019	0.001	0.012	0.026	0.001	ns
72	D2_101			0.004	0.028	0.025	0.066	0.085	0.011	ns
73	D2_110			0.000	0.010	0.001	0.015	0.016	0.001	ns
74	D2_122			0.012	0.012	0.113	0.019	0.059	0.057	ns
75	D2_129			0.012	0.003	0.036	0.061	0.020	0.086	ns
76	D2_149			0.009	0.004	0.000	0.025	0.006	0.011	ns^*^
77	D2_156			0.020	0.009	0.038	0.022	0.070	0.004	ns
78	D2_160			0.008	0.018	0.069	0.002	0.017	0.032	ns
79	D2_176			0.001	0.005	0.056	0.042	0.030	0.070	ns
80	D2_216			0.005	0.051	0.026	0.022	0.007	0.009	ns
81	D2_247			0.000	0.029	0.001	0.036	0.036	0.001	ns
82	D2_265			0.030	0.010	0.024	0.015	0.004	0.001	ns
83	D2_266			0.001	0.019	0.049	0.099	0.123	0.034	0.020^*^
84	D2_267			0.031	0.009	0.090	0.049	0.147	0.017	ns
85	D2_276			0.005	0.000	0.001	0.012	0.002	0.011	ns
86	D2_281			0.000	0.004	0.011	0.025	0.026	0.011	ns
87	D2_283			0.000	0.000	0.001	0.000	0.000	0.001	ns
88	D2_284			0.000	0.004	0.011	0.025	0.026	0.011	ns
89	D2_298			0.005	0.040	0.010	0.031	0.012	0.001	ns
90	D2_332			0.051	0.001	0.011	0.028	0.007	0.020	ns
91	D2_339			0.002	0.000	0.001	0.008	0.002	0.006	0.000^*^
92	D2_342			0.000	0.002	0.006	0.001	0.002	0.005	ns
93	D2_357			0.000	0.000	0.001	0.000	0.000	0.001	ns
94	D2_380			0.009	0.000	0.005	0.000	0.007	0.001	ns^*^
95	D3_068			0.013	0.019	0.001	0.000	0.013	0.019	0.000^*^
96	D3_070			0.007	0.006	0.059	0.057	0.102	0.026	ns
97	D3_079			0.033	0.000	0.001	0.030	0.000	0.026	ns
98	D3_106			0.083	0.003	0.013	0.021	0.027	0.037	ns
99	D3_113			0.000	0.031	0.055	0.006	0.007	0.054	ns
Mean (146 loci)				0.035	0.028	0.064	0.082	0.085	0.070	
100	D3_156			0.000	0.000	0.001	0.000	0.000	0.001	ns
101	D3_166			0.000	0.010	0.001	0.015	0.016	0.001	ns
102	D3_201			0.010	0.000	0.055	0.028	0.059	0.025	ns
103	D3_224			0.034	0.049	0.068	0.020	0.002	0.007	ns
104	D3_241			0.022	0.036	0.005	0.015	0.061	0.006	ns^*^
105	D3_256			0.007	0.000	0.003	0.001	0.002	0.001	ns
106	D3_258			0.000	0.009	0.024	0.047	0.052	0.020	ns
107	D3_261			0.006	0.006	0.028	0.027	0.008	0.057	ns
108	D3_304			0.003	0.001	0.012	0.015	0.006	0.024	ns
109	D3_305			0.052	0.000	0.013	0.011	0.017	0.015	ns
110	D3_306			0.000	0.009	0.011	0.000	0.000	0.011	ns
111	D3_343			0.022	0.002	0.033	0.081	0.021	0.101	ns
112	D3_370			0.038	0.000	0.031	0.000	0.035	0.001	ns^*^
113	D3_385			0.016	0.049	0.031	0.006	0.002	0.088	ns
114	D4_082			0.081	0.047	0.022	0.006	0.129	0.019	ns
115	D4_085			0.033	0.010	0.001	0.005	0.016	0.026	ns
116	D4_094			0.001	0.010	0.000	0.021	0.012	0.003	ns^*^
117	D4_105			0.000	0.028	0.025	0.084	0.085	0.024	ns
118	D4_108			0.017	0.000	0.031	0.086	0.035	0.081	ns
119	D4_111			0.003	0.000	0.001	0.000	0.002	0.001	0.000^*^
120	D4_130			0.023	0.036	0.003	0.000	0.020	0.039	ns
121	D4_140			0.016	0.009	0.024	0.108	0.052	0.071	0.001^*^
122	D4_150			0.003	0.008	0.039	0.025	0.013	0.054	ns
123	D4_153			0.013	0.027	0.016	0.031	0.082	0.000	ns
124	D4_154			0.016	0.000	0.001	0.025	0.002	0.024	ns
125	D4_156			0.004	0.005	0.004	0.005	0.000	0.000	ns
126	D4_175			0.016	0.000	0.010	0.000	0.013	0.001	ns^*^
127	D4_181			0.013	0.000	0.001	0.022	0.002	0.019	ns
128	D4_183			0.003	0.011	0.005	0.053	0.031	0.016	ns
129	D4_185			0.009	0.001	0.005	0.015	0.001	0.024	ns
130	D4_194			0.069	0.004	0.030	0.001	0.057	0.008	ns
131	D4_195			0.017	0.000	0.001	0.015	0.000	0.012	ns
132	D4_196			0.000	0.010	0.001	0.015	0.016	0.001	ns
133	D4_204			0.013	0.010	0.001	0.002	0.006	0.019	ns
134	D4_206			0.027	0.001	0.084	0.028	0.096	0.020	ns
135	D4_227			0.001	0.010	0.010	0.001	0.000	0.006	ns^*^
136	D4_229			0.004	0.008	0.025	0.000	0.007	0.011	ns
137	D4_232			0.003	0.031	0.090	0.036	0.022	0.108	ns
138	D4_257			0.030	0.001	0.006	0.005	0.012	0.011	ns
139	D4_283			0.023	0.040	0.017	0.047	0.007	0.001	0.000^*^
140	D4_340			0.000	0.009	0.011	0.000	0.000	0.011	ns
141	D4_360			0.000	0.022	0.039	0.098	0.099	0.039	ns
142	D4_361			0.010	0.019	0.001	0.005	0.026	0.006	ns
143	D4_363			0.003	0.000	0.001	0.000	0.002	0.001	ns^*^
144	D4_366			0.030	0.018	0.017	0.031	0.000	0.003	0.024^*^
145	D4_398			0.000	0.027	0.011	0.059	0.059	0.011	ns
146	D4_404			0.019	0.000	0.039	0.008	0.043	0.006	ns^*^
Mean (146 loci)				0.035	0.028	0.064	0.082	0.085	0.070	

**Table 2:** Percentage of population-differentiating loci (type p, Fig. 2) by population, in comparison with all scored loci (scored) and informative loci (used)

Population	*n* Loci	% Used loci	% Scored loci
aga-1	6	4.1	1.7
aga-3	7	4.8	2.0
pha-1	3	2.1	0.9
pha-3	8	5.5	2.3
Mean	6	4.1	1.7

**Table 3:** Percentage of loci falling into categories as mentioned in Figure 2, and percentage of loci in these categories deviating significantly from neutral expectations according to introgress results for population hyb-1; in comparison with all scored loci (scored) or only informative loci (used)

Type	*n* Loci	% Loci		% Skewed
		Used	Scored	
s	20	13.7	5.8	30.0
s^h^	11	7.5	3.2	45.5
l	2	1.4	0.6	50.0
p	24	16.4	7.0	–
p^1^	9	6.2	2.6	22.2
x	23	15.8	6.7	13.0
None (used)	77	52.7	22.4	10.1
Total (used)	146	100.0	42.4	–
None (scored)	275	–	79.9	2.9
Total (scored)	344	–	100.0	–

1 Only using loci from the sympatric parental populations (aga-1, pha-1), as a skew for aga-3/pha-3 cannot be assessed due to the lack of hybrids.

### Genomic clines (introgress)

#### Datasets (hyb-1) vs. (hyb-1 and hyb-2)

The clines estimated for all hybrids (hyb-1 and hyb-2 together) and only the strictly sympatric hybrids (hyb-1) qualitatively agreed; apart from one exception all loci that were significant for hyb-1 were also significant for hyb-1 and hyb-2 combined. However, ten loci for which clines did not show a significant (*P* < 0.05) deviation from neutral expectations only using the data from hyb-1, did so in the combined dataset (40, 76, 94, 104, 112, 116, 126, 135, 143, 146; Table1), and one locus showing significant deviation in hyb-1 was not significant for the combined data (36; Table1). Because hyb-2 showed a significant bias toward a hybrid index of 0.5, apparently lacking backcrosses (Fig. 7), and as the Structure results indicated that this population might have an unusual structure, only clines showing significant deviation in hyb-1 were considered for further discussion, in attempt to minimize influence from stochastic processes, that were likely to have affected an inbred later-generation hybrid population. Due to the restricted range of hybrid indices in hyb-2, a separate cline analysis for this population only was not possible.

#### Deviation by locus category

Of the loci that showed genomic clines significantly deviating from neutral expectations in hyb-1, six were type s loci (five of these s^h^), four type p (two differentiating aga-1 and two aga-3), one a type l locus, three of type x, and seven uncategorized. With regard to all analyzed loci, the following percentage of loci in each category showed clines deviating significantly from neutral expectations: 45% of type s^h^ loci (30% for type s overall), 50% of type l loci (one locus of two), 22% of type p loci that differentiated aga-1 and pha-1, 13% of type x, and 10% without category (Table3).

#### Patterns of significant clines

**Figure 6:**
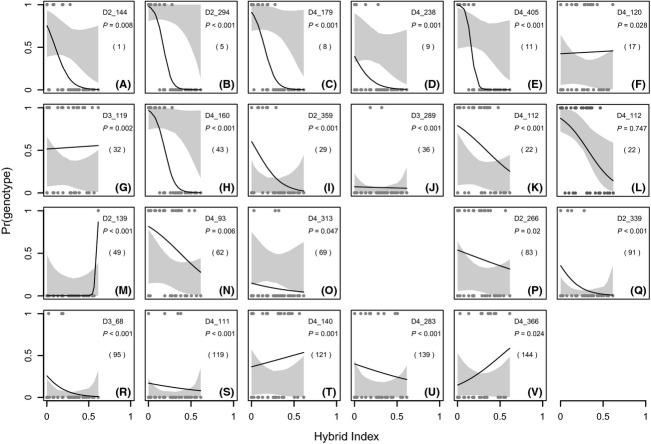
Genomic clines of loci deviating significantly (*P* < 0.05) from neutral expectations in hyb-1, as estimated with the R package introgress. Confidence intervals for neutral clines (gray areas) and observed clines (black lines) are shown. The dominant genotype observed in individuals is indicated by the points at top and bottom. A hybrid index of 0 indicates a genomic composition as of *Rhododendron aganniphum*, and an index of 1 as of *R. phaeochrysum*. On the right side of each subgraphic are given the original locus name, the *P*-value for deviation from neutral expectations (based on 10,000 replications), and in brackets the corresponding locus number in Table1. (A–E) type s^h^ loci; (F) type s locus; (G, H) type p loci, differentiating for one of the populations sympatric with the hybrids (aga-1); (I, J) type p loci, differentiating for one of the populations not sympatric with the hybrids (aga-3); (K, L) type l locus: the deviation of the cline from neutrality was significant for the original data (K); however, when assuming that the fragment in aga-1 was not homologous to the one in pha-1, the cline showed no deviation; (M–O) type x loci; (P–V) loci without category.

All clines of type s^h^ showed a very clear pattern, where individuals with a hybrid index close to 0.5 all had the same allele (Fig. 6A–E, dots at the bottom), with the clines approaching expectations toward a hybrid index of 0 (backcrossing toward *R. aganniphum*). This same pattern was also observed in one of the type p loci which showed exceptional differentiation between aga-1 and pha-1 (Table1, 43; Fig. 6H). The significant type s locus (17) and the second type p locus for which aga-1 and pha-1 were differentiated (32) shared the quality of this pattern – significant deviation close to a hybrid index of 0.5 and approaching expectations toward an index of 0 – but not as strongly pronounced (Fig. 6F,G). The cline in the only significant type l locus showed an inverted pattern instead, where close to a hybrid index of 0.5, the observed cline was within the confidence interval for neutrality, but deviated in backcrosses toward *R. aganniphum* (Fig. 6K). At this locus, the population pha-1 had a very high frequency of the dominant allele, which was at very low frequency in pha-3 and nearly absent in aga-3; aga-1 showed high frequency but significantly lower than pha-1. The significance of the cline was due to the hybrids showing frequency of the null allele even higher than aga-1. Hence, the cline pattern did not conform to any biological explanation. The only sensible explanation seemed to be that the fragment in aga-1 had the same length, but was not homologous to the one in pha-1, a problem that seems to occur frequently with AFLPs (Bonin et al. 2007). The only way to confirm this would have been sequencing of the fragments, which was not possible when the potential problem was noticed. Therefore, an additional cline analysis was performed for which the frequency of the dominant allele in aga-1 was set to 0, thereby assuming aga-1 was lacking the allele present in the pha-1 population. In this case, the observed cline did not show significant deviation (Fig. 6L). Two of the four significant type p loci (Table1, 29, 36) differentiated aga-3 from the other populations, so that aga-1 and pha-1 showed no allele frequency differences for those loci, and hence, no deviation in the hybrids would have been expected. One of those loci might indeed be a false positive, as it was the only locus significant in hyb-1 but not in the combined dataset (hyb-1 and hyb-2), and additionally, its cline showed no clear pattern (Table1, 36; Fig. 6J).

The second of those two loci, however, shows a pattern similar to that of the type l locus, and not similar to the other type p loci, which differentiate aga-1 and pha-1 (Fig. 6I).

The three significant type x loci and the seven significant unclassified loci show a mixture of the two aforementioned patterns, but in all cases, less pronounced; an exception might be locus 49 for which nearly all hybrids have the same allele (Fig. 6L).

#### Simulated hybrids

In the simulated hybrid populations, very few loci showed clines deviating from neutral expectations, even when backcrosses to pha-B were removed. Seven loci showed significantly deviating clines in one of the ten simulated datasets (loci 46, 52, 73, 112, 126, 132, and 139). Two further loci showed significant deviation in several: locus 28 (D2_313) in six of the ten simulated datasets and locus 59 (D3_259) in all ten. Apart from locus 46, all of these loci fall into category x or into no category, and in no case was a locus significant that was significant for the original data.

## Discussion

The two hybrid zones show very divergent patterns of composition; one potentially allows unidirectional gene flow, while the other presents a barrier. While most of the genome is unaffected by incompatibilities, nearly half of species-differentiating markers show transmission ratio distortion in hybrids. Transmission ratio distortion is already visible in F1 individuals; therefore, most incompatibility factors are likely to be still segregating in the species.

### Inherent bias in the data

Before discussing the results from the original data, it is important to highlight some findings of the simulated hybrid populations. As the parental populations for these simulations were themselves generated, they conformed to the assumptions of no linkage between loci and were in Hardy–Weinberg equilibrium. Hence, it is reasonable to assume that any observed biases were due to peculiarities in parental allele frequencies, combined with the dominant nature of the data and how the programmes used treated these. While the assignment of the hybrids by STRUCTURE was generally as expected, and hybrids showed clear admixture from both parents (Fig. 5A), estimates of *Q* were significantly biased toward the *R. aganniphum* cluster; this was apparent in the F1s as well as the backcrosses to *R. aganniphum* (Fig. 4; F1, BC1A, BC2A). The strength of this bias suggested that even early-generation backcross individuals to *R. aganniphum* would probably be indistinguishable from pure *R. aganniphum*, as the backcrosses showed a *Q* for the aga-A cluster above 0.95, thereby being comparable to pure individuals when taking noise in the data into account. Interestingly, backcrosses toward *R. phaeochrysum* did not show any significant bias (Fig. 4; BC1B, BC2B), so that these hybrids should have been detectable.

The hybrid index of introgress was less affected (Fig. S4), the bias being mostly visible in the F1 generation (mean of 0.45), but not being significant in backcrosses. Therefore, the cline analyses should not be affected significantly by this pattern.

### Hybrid zone composition

When analyzed, the sympatric population (hyb-1) and the parapatric population (hyb-2) showed different patterns of hybridization, which agreed with initial morphological observations.

#### Baima Shan (hyb-2)

**Figure 7:**
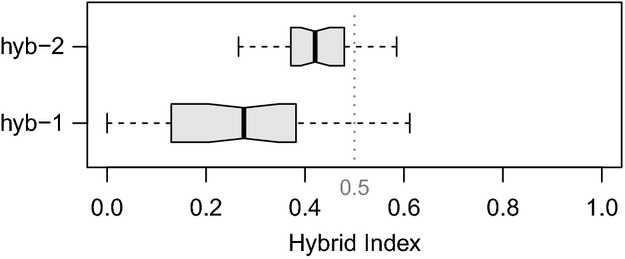
Boxplot of observed hybrid indices in the two hybrid populations hyb-1 and hyb-2. Hybrid indices were obtained with introgress, and an index of 0 indicates genomic origin from *Rhododendron aganniphum*, and 1 from *R. phaeochrysum*.

The isolated hybrid population hyb-2, which was characterized by uniform morphology, showed a hybrid index between 0.4 and 0.5 (Fig. 7), which, when taking the above-mentioned bias of the data into account, suggested a population comprising mostly or only F1 hybrids, a situation relatively common in *Rhododendron* (Milne et al. 2003; Milne and Abbott 2008; Zha et al. 2010). However, the clustering of STRUCTURE did not entirely conform with this scenario, as the hybrids formed their own cluster (Fig. 3B), while the simulations suggested that F1s should show admixture (Fig. 5A,B). Only populations comprising inbred later-generation hybrids showed the same tendency to form a separate cluster as observed for hyb-2. However, for all these populations, *K* = 2 was always the most likely number of clusters, and hybrids showed more equal admixture proportions from the parents than observed in hyb-2 (Fig. 3A). Therefore, it seems likely that the transmission distortion of several highly differentiated markers (e.g., type s^h^ loci) contributed to the observed pattern. Due to the nature of the data, it is not possible to distinguish between a scenario of mostly F1 hybrids or a swarm of inbred later-generation hybrids. F1 hybrids could show particular allele frequencies due to strong transmission distortion, which could, for example, be caused by lethality of certain allele combinations at loci that are polymorphic in the parents, or ecological selection eliminating F1 hybrid offspring that lack the right combination of alleles. On the other hand, the apparent scarcity of individuals from either parental species in the vicinity of hyb-2 could have led to the establishment of an inbreeding hybrid swarm comprising later-generation hybrids. Although this distinction is not possible, the data give strong evidence that backcrosses to either parent are absent from hyb-2, suggesting that this hybrid zone does not contribute to gene flow between *R. aganniphum* and *R. phaeochrysum*.

#### Baima Shan (hyb-1)

The other hybrid population, hyb-1, was sympatric with both parents and diverse in both morphology and marker profiles (Fig. 3B), showing a wide range of hybrid indices between 0 and 0.6 (Fig. 7), suggesting segregating hybrid derivatives with a large proportion of backcrosses, many of them later generation. That many of the morphologically identified hybrids in hyb-1 showed extraordinarily high admixture proportions from the *R. aganniphum* cluster (Fig. 3A,B) is likely to be due to the inherent bias in the data. Furthermore, the simulations showed that backcrosses to *R. phaeochrysum* should have the expected admixture proportions; hence, admixture in pha-1 from the hybrid cluster (Fig. 3B) is more likely to be indicative of an artifact than introgression. This artifact behavior is also suggested by the increased *Q* for the hybrid cluster of some individuals in aga-1 when comparing *K* = 2 to *K* = 3 (Fig. 3). Therefore, there is good evidence that backcrossing in hyb-1 is entirely asymmetrical toward *R. aganniphum*.

Asymmetries in crossing barriers in angiosperms are widespread and can occur during several stages before seed development (Tiffin et al. 2001; Turelli and Moyle 2007). In *Rhododendron*, phenology has been suggested in certain cases as an explanation for asymmetric backcrossing (Milne and Abbott 2008). This, however, probably plays a minor role in the case of *R. aganniphum* and *R. phaeochrysum*, as the flowering times of both species have a large overlap and pollinators do not discriminate between flowers (T. Marczewski, pers. obs.). Pollen competition might contribute, but on its own should allow a few backcross events toward *R. phaeochrysum*, as mostly paternity bias would be expected (Emms et al. 1996; Rahmé et al. 2009). Simple intrinsic allelic incompatibilities as, for example, Bateson–Dobzhansky–Muller incompatibilities (BDMI), should theoretically not lead to asymmetries in reproductive isolation (Tiffin et al. 2001). However, more complex cases including few BDMIs of large effect, and most importantly involving interactions at reproductive stages which can be expected to have highly asymmetric parental contributions due to differences in ploidy can apparently lead to asymmetric barriers (Turelli and Moyle 2007). In angiosperms, different ploidy levels are, for example, realized in pollen–pistil interactions (haploid–diploid), or in interactions between the embryo and the endosperm (diploid–tetraploid/polyploid). Furthermore, in some cases, these asymmetric barriers have been shown to be very strong, even between closely related species, and are able to lead to complete isolation in one direction (Turelli and Moyle 2007). Therefore, the observed pattern in hyb-1 suggests that unidirectional gene flow from *R. phaeochrysum* into *R. aganniphum* is likely, but that the hybrid zone does not provide a bridge for introgression into *R. phaeochrysum*.

#### Daxue Shan (aga-3 and pha-3)

At Daxue Shan, where the populations aga-3 and pha-3 were collected, no individuals were observed that showed any indication of hybrid morphology. That hybrid individuals were overlooked was one possibility, as the population was covering a large area. However, hybrid zones between the two species normally comprise a considerable number of intermediates, and the zone in which both species met was searched intensively, making this scenario unlikely. Furthermore, admixture proportions from the respective other cluster for the four admixed parental individuals were substantial (*Q* for the respective other cluster above 0.3; Fig. 3), suggesting that at least in the admixed individuals from aga-3, some hybrid morphology should have been apparent, if the individuals were early-generation hybrids. The two other possibilities are that hybrids never existed at the locality or that hybrids existed in the past, but are not longer present. In the latter case, one might expect to find some remnants of introgression, and the STRUCTURE results strongly suggested admixture for three individuals of aga-3 and one individual of pha-3 (Fig. 3). Therefore, it seems likely that at some point in the past, hybrids were present, and if the hybrid zone composition was similar to that of hyb-1, higher levels of introgression into aga-3 would be expected. The occurrence of the admixed pha-3 individual, however, suggests that introgression does not have to be entirely asymmetric in all settings where the species hybridize.

### Permeability of the genome

The analysis of the hybrid population hyb-1 provided evidence for considerable potential for gene flow in certain settings, and a history of introgression seems likely for both parental populations. Of the 344 scored markers, 21 showed clines significantly deviating from neutral expectations (Fig. 6). However, only six of these 21 loci showed clines strongly indicative of being affected by a barrier (Fig. 6A–E,H); hence, 338 of 344 loci (98%) seem to have the potential to be more or less affected by introgression. Large parts of the genomes of the species could therefore have experienced episodes of gene flow contributing to low differentiation for most loci.

The distribution of pairwise *F*_ST_ values for all comparisons showed a marked L-shape, due to the strong bias toward low values (Fig. S5). This is a pattern that has been previously reported in other closely related hybridizing species such as *Silene* (Minder and Widmer 2008) and *Helianthus* (Yatabe et al. 2007), but has also been observed in very early stages of speciation, as between ecotypes in *Helianthus* (Andrew and Rieseberg 2013) and host races in pea aphids (Via 2009). In the tail of this L-shaped distribution are markers that show relatively high species differentiation, which amounts to 3.2% of loci in this study (Table3). Hence, more loci show significant differentiation between species than seem to be affected by incompatibilities. This suggests that opportunities for gene flow were not as frequent in the past as to prevent differentiation from arising in parts of the genome that should be permeable to gene flow. Although several loci that show neutral clines in hybrids are differentiated between species, a disproportionately large number of loci showing high differentiation (type s^h^) deviate significantly from expectations (45%, Table3). This observation conforms to predictions by the genic species concept (Wu 2001), as parts of the genome harboring incompatibilities or environmentally selected genes are expected to experience substantially reduced gene flow, which would allow neutral mutations in these regions to increase in frequency undisturbed by introgression from alleles from the other species.

Interestingly, the cline patterns for all s^h^ loci were similar, and very clear, with all individuals with a hybrid index close to 0.5 only having one type of allele (Fig. 6A–E). For the hybrid population hyb-2, the identity of hybrids close to 0.5 is uncertain; however, in hyb-1, it seems most likely that these are F1 hybrids, as many backcrosses are present, and it is less parsimonious to assume that inbred later-generation hybrids had to arise before backcrossing became possible. This scenario is made even less likely by the observation that hybrids with a hybrid index close to 0.5 are rather scarce in hyb-1. Therefore, the observed cline patterns suggest that selection, leading to a transmission ratio distortion, already acted at the F1 stage, as all of these hybrids only show one type of allele. This behavior can be expected if hybrid incompatibility factors are still segregating in the parental populations (Maheshwari and Barbash 2011), which seems probable as no fixed differences between the species were observed. If the majority of loci responsible for incompatibilities were polymorphic, it would suggest that the strongest reproductive barrier should be the formation of F1 hybrids, while later generations would be less affected, as the viable F1s would mostly have a compatible composition of alleles.

AFLPs are expected to be more or less randomly distributed throughout the genome (Bonin et al. 2007), and background linkage in the species can be expected to be low (outcrossing, long-lived species). Therefore, the proportion of species-differentiating markers should give a reasonable estimate for the genome. However, the dominant nature of the data did not allow an estimate of linkage between markers, and hence, it is not clear whether s^h^ loci showing a similar pattern are distributed throughout the genome or occur in linked clusters, which has been reported before for loci affected by transmission ratio distortion (Tang et al. 2010).

### Cline patterns in loci that show population differentiation within species

#### Type p loci

Four population-differentiating loci (type p) and one locality-differentiating locus (type l) showed clines significantly deviating from neutral expectations. Two of the significant type p loci (29 and 36) differentiated aga-3 from the other populations, while aga-1 and pha-1 showed no differentiation. As there is no information about how these loci would behave in hybrids with aga-3 as one parent, it seems unreasonable to make inferences of what caused the significant deviation. For the other two type p loci (32 and 43), the population aga-1 was differentiated from the other populations and hence the sympatric parents (aga-1 and pha-1). The clines of both of these loci showed a pattern similar to the significant type s loci (Fig. 6G and H), indicating that they were affected by incompatibilities. This pattern could have be caused in two different ways. One possibility is that the incompatibility affecting the locus existed in *R. aganniphum* in general before the mutation occurred in aga-1. In this case, the new allele could have been able to rise to a higher frequency because it was protected from gene flow, while at the same time, gene flow between the populations aga-1 and aga-3 was not high enough for the allele to spread. The other possibility is that an incompatibility arose in aga-1, which is not present in aga-3, thereby protecting the linked locus from gene flow and allowing higher differentiation in aga-1 as opposed to aga-3. It has been suggested that advantageous mutations are likely to spread relatively quickly within a species metapopulation (Morjan and Rieseberg 2004). The observed *F*_ST_ between aga-1 and aga-3 at least for locus 43 is, however, higher than most type s loci (*F*_ST_ = 0.454, Table1), suggesting that no gene linked to the locus actually confers an adaptive advantage. Furthermore, theory suggests that in light of gene flow, the maintenance of incompatibilities, at least BDMIs, is more likely when the incompatibilities additionally convey an adaptive advantage (Bank et al. 2012). Therefore, theory would give slight preference to the scenario of a neutral mutation having arisen in linkage to an existing incompatibility locus, as a newly arisen incompatibility locus would probably have spread, carrying a linked allele with it. However, to answer this question, one would have to identify the nature of the incompatibility loci affecting the two type p loci.

#### Type l locus

The *F*_ST_ patterns for the only significant type l locus (22) showed very high differentiation between both localities (Baima Shan, aga-1 and pha-1, and Daxue Shan aga-3 and pha-3 (Table1, 22)). As this was unusual, the most likely explanation seemed to be introgression of the dominant pha-1 allele into the aga-1 population, which would be plausible due to a high number of backcrosses allowing gene flow in this direction. In this case, it would have been expected that the cline deviated because of a higher than expected frequency of the dominant allele in the hybrids, causing the cline to be at the bottom of the graph (Fig. 6K). This was, however, not observed, and the significant deviation of the cline was instead caused by a prevalence of the recessive allele in the hybrids, even higher than in aga-1. Hence, the patterns were conflicting and did not lend themselves to a plausible biological scenario. One possible explanation for the pattern seemed to be that the AFLP fragment present in aga-1 was not homologous to the fragment in pha-1, or indeed the one in the hybrids, because if the hybrids would have contained the fragment, no deviation should have resulted. Testing this possibility by setting all dominant phenotypes in aga-1 for this locus to 0, thereby assuming aga-1 was lacking the fragment, led to a neutral cline for the locus (Fig. 6L). As the presence of a nonhomologous fragment in aga-1 seems the best explanation for the observed pattern, it is most likely that locus 22 is rather population-differentiating for pha-1, showing a cline that can be expected under neutrality, and not a locality-differentiating locus. In this case, the data does not provide good evidence for the existence of loci showing differentiation by locality between the populations.

## Conclusions

The two analyzed hybrid populations showed a very different composition of admixed individuals. In the morphologically diverse hyb-1, entirely asymmetric backcrossing to *R. aganniphum* was prevalent, thereby allowing unidirectional gene flow from *R. phaeochrysum* to *R. aganniphum*. Contrastingly hyb-2 comprised either F1s, later-generation hybrids, or a mixture of both, but no backcrossed individuals to either of the parental species, hence not providing a bridge for gene flow. Admixture of some individuals in the populations aga-3 and pha-3 suggested that hybrids were once present at Daxue Shan, but are absent in the current population. A large proportion of the genome did not appear to be affected by incompatibilities, and up to 98% of loci are potentially not protected from introgression. In contrast, nearly half of the markers that showed high species differentiation seemed to be linked to a locus causing transmission ratio distortion in early-generation hybrids. Therefore, most factors conveying incompatibilities are likely to be still segregating within species. On the other hand, over half of species-differentiating markers did not show unusual cline patterns in the hybrid population, indicating that the species have accumulated neutral mutations in parts of the genome that are not affected by loci causing incompatibilities.

Overall, observed patterns conformed to predictions made by the genic species concept, as parts of the genome seemed to be affected by incompatibilities affecting gene flow between species, while other parts do not show such patterns. A strong population-specific genetic component influencing cross-compatibility was not implied by the data, as it seemed more likely that population-differentiating loci experiencing transmission ratio distortion are located within areas of the genome generally affected by incompatibilities. Furthermore, after correcting for experimental artifacts, there seemed to be no clear evidence for preferential introgression of any population-specific marker. The absence of a clear population-specific genetic component influencing hybridization suggests that environmental conditions play a major role in influencing the occurrence of hybrids as well as hybrid swarm composition. Incompatibilities seemed to be affecting the generation of F1 hybrids most; hence, it is plausible that they are only formed if environmental conditions tip the balance in their favor. This might only happen sporadically, but as both species are long-lived, the hybrids would remain present in the population for a long time. This could explain the complete absence of hybrids at Daxue Shan, which seemed ecologically similar to Baima Shan; the conditions could have been different when most of the initial hybrids where formed.

## Data Accessibility

The original AFLP scoring matrix along with all R scripts and custom functions used for data handling and analyses together with the STRUCTURE input files are available on Dryad ( http://dx.doi.org/10.5061/dryad.k53g5 )

TM, DC, and RM conceived the study and carried out fieldwork. TM conducted experiments and analyzed data. TM and RM wrote the paper.
