# Supplementary material for: Hybridization in closely related Rhododendron species: half of all species-differentiating markers experience serious transmission ratio distortion
Source: Ecol Evol. 2015 Jul 7;5(15):3003–22. doi: 10.1002/ece3.1570 (PMC4559045; doi:10.1002/ece3.1570)
Supplement: Supplementary file 1 [file ece30005-3003-sd1.pdf]

Supplementary Material for:

Hybridisation in closely related *Rhododendron* species: half of all species differentiating markers experiance serious transmission ratio distortion.

T. Marczewski, D.F. Chamberain, R.I. Milne

Loci categories: population differentiating loci (type p)

The locus category of type p was based on considerations of extreme cases that can occur along a continuum which has at one end the case where one population is clearly differentiated from all others, and on the other end the case where most populations have intermediate allele frequencies, not implying a clear pattern of differentiation.

When using F_{ST}-values to determine the level of differentiation, the clear case should show a equally high F_{ST} for all pairwise comparisons involving the population in question, while all other pairwise comparisons should be close to 0. Normalising by dividing by the highest F_{ST} should then lead to all pairwise comparisons involving the population being ≈ 1.

For four populations we have six pairwise comparisons, three involving the focal population, and a further three between the other three populations. Grouping the F_{ST}-values after normalisation and using (a) to denote comparisons involving the focal population, and (b) for the other comparisons, we get: $a\,(1,1,1) \quad mean = 1$ and $b\,(0,0,0) \quad mean = 0$
Hence the difference between the means for this clear case is $1 - 0 = 1$.

If the population is less clearly differentiated from the other populations, the difference between F_{ST}-values involving the focal population and F_{ST}-values not involving the focal population should become smaller, and hence the means of normalised F_{ST}-values will approach each other, leading to a smaller difference. This can be exemplified with some special cases involving different patterns of differentiation.

In the next case one population (not the focal one) has an intermediate allele frequency, and therefore F_{ST}-values involving this population will be between the maximum F_{ST} and the minimum F_{ST}, or for simplicity ≈ 0.5 after dividing by the highest F_{ST}. In total three pairwise comparisons will involve this intermediate population, one of these with the focal population, and two with the other two non-focal populations, resulting in:

$$\left.\begin{array}{ll} a\,(1,1,0.5) & mean = \frac{2.5}{3} \\ b\,(0.5,0.5,0) & mean = \frac{1}{3} \end{array}\right\} \frac{2.5}{3} - \frac{1}{3} = 0.5$$

For the last case we consider two populations with intermediate frequencies, again none of them is the focal population. For simplicity we also assume exact intermediacy leading to a normalised F_{ST} of 0.5. However, as now two populations have intermediate allele frequencies the pairwise F_{ST} for this comparison will be ≈ 0, while all other comparisons will have a higher F_{ST}, giving:

$$\left.\begin{array}{ll} a\,(1,0.5,0.5) & mean = \frac{2}{3} \\ b\,(0.5,0.5,0) & mean = \frac{1}{3} \end{array}\right\} \frac{2}{3} - \frac{1}{3} = 0.33$$

Proceeding further down this route will end with the case of all populations having intermediate frequencies, and hence the pairwise F_{ST}-values of all comparisons would be similar, with the difference between normalised means approaching 0.

The difference between means of normalised F_{ST}-values that involve the focal population and that do not involve the focal population, can therefore range from 1 to 0, where 1 represents the clear cut case with the focal population being differentiated from all others, and 0 represents no differentiation between any of the populations. Up to a value of 0.5 the population is more differentiated from two of the other populations, and from a value of 0.33 the situation becomes complex, with no easy interpretation of differentiation patterns. These values represent theoretical cases in a continuum, and are mostly not found in real data. Especially the clear cut case is difficult to realise as slight deviations in allele frequencies lead to a fast drop of the index below 1. However, already values above 0.5 mean that the only intermediate population is approaching the allele frequencies of the other non-focal populations, indicating the more differentiated status of the focal population. As a value of 1 is barely ever observed, a cutoff value has to be chosen that is a compromise between a pattern indicating differentiation of the focal population from the others, but allowing for certain allele frequency differences between the populations. This cutoff should clearly be between 1 and 0.5, as above 0.5 the focal population becomes increasingly differentiated from the other three. To mirror the behaviour below a value of 0.5, where differentiation patterns become unclear around 0.33, a cutoff value of $1 - 0.33 = 0.67$ was used.

Structure ΔK

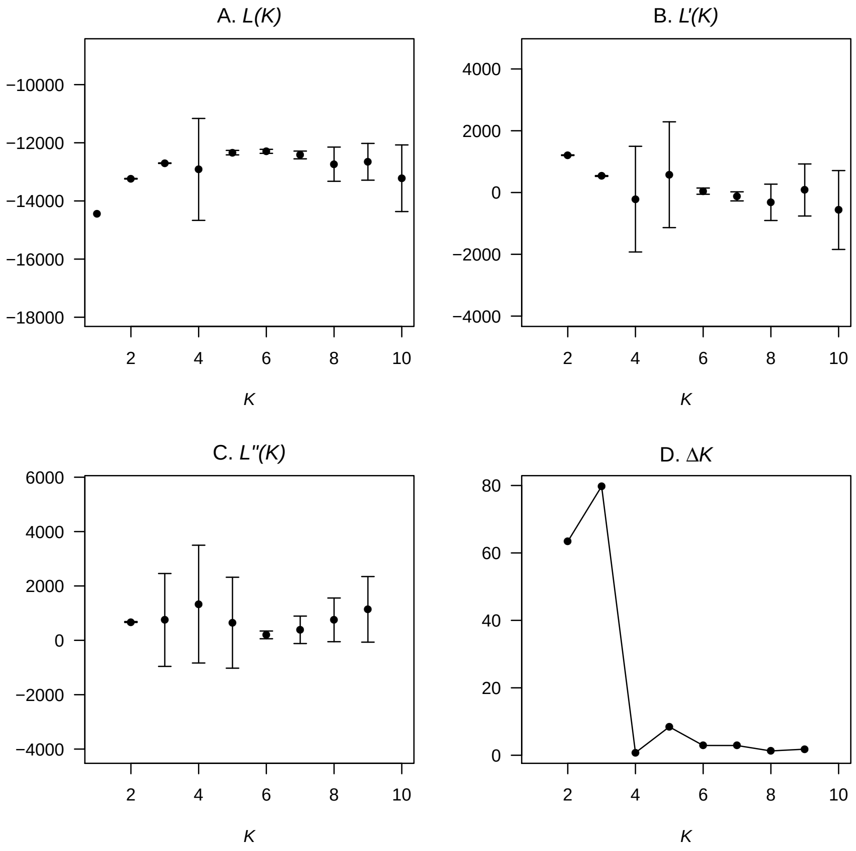

Supp_Figure 1a. Structure analysis with all populations included (aga-1, aga-3, pha-1, pha-3, hyb-1, and hyb-2); 20 runs per K were performed. Plots of the log likelihood of ten structure runs for each K, and differentials thereof, to obtain ΔK as an indicator of the most likely number of clusters in the data, as suggested by Evanno *et al.* (2005).

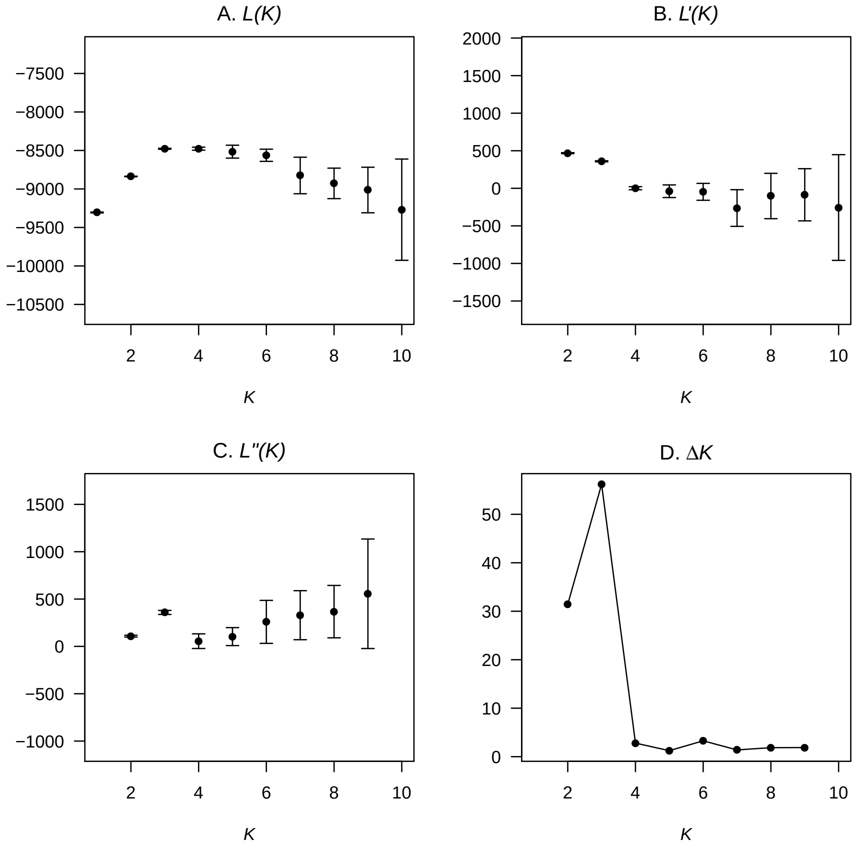

Supp_Figure 1b. Structure analysis with only sympatric parents (aga-1, pha-1) and hybrids (hyb-1, hyb-2) included; 20 runs per K were performed. Plots of the log likelihood of ten structure runs for each K, and differentials thereof, to obtain ΔK as an indicator of the most likely number of clusters in the data, as suggested by Evanno *et al.* (2005).

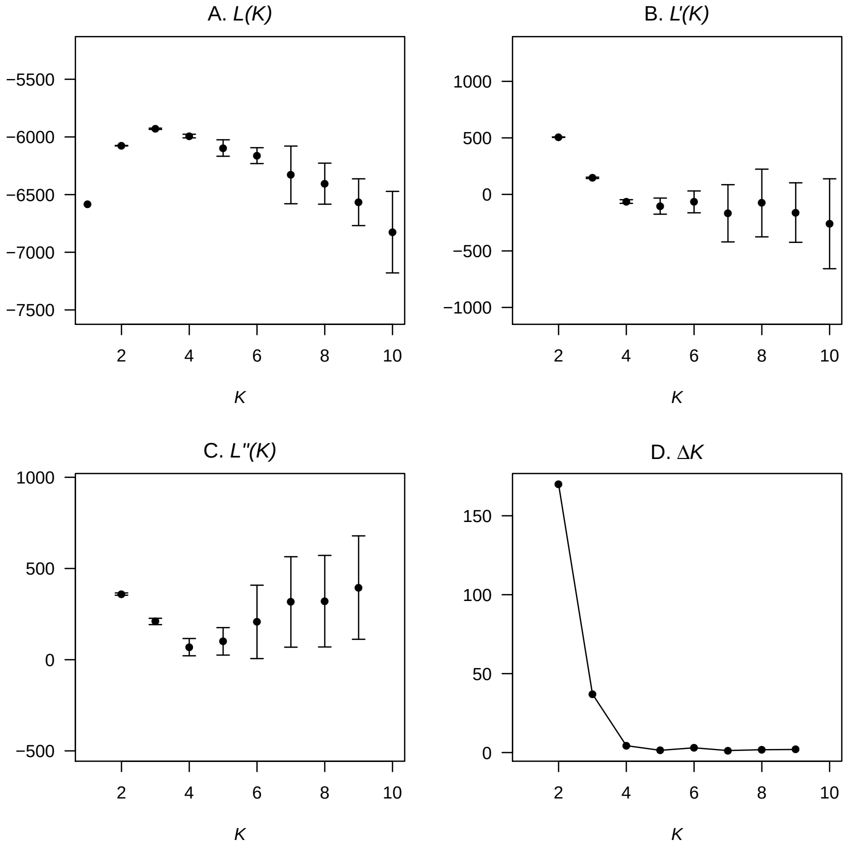

Supp_Figure 1c. Structure analysis with only sympatric parents (aga-1, pha-1) and the hybrid population hyb-1 included; 20 runs per K were performed. Plots of the log likelihood of ten structure runs for each K, and differentials thereof, to obtain ΔK as an indicator of the most likely number of clusters in the data, as suggested by Evanno *et al.* (2005).

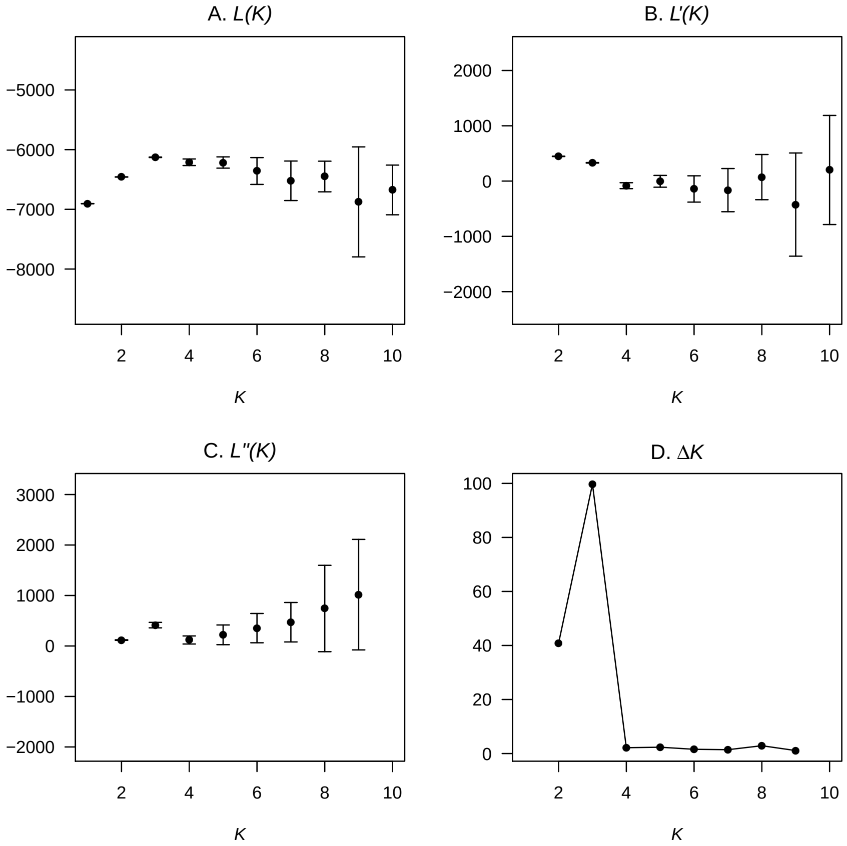

Supp_Figure 1d. Structure analysis with only sympatric parents (aga-1, pha-1) and the hybrid population hyb-2 included; 20 runs per K were performed. Plots of the log likelihood of ten structure runs for each K, and differentials thereof, to obtain ΔK as an indicator of the most likely number of clusters in the data, as suggested by Evanno *et al.* (2005).

Introgress: clines of all loci

Pooled hybrid populations hyb-1 and hyb-2

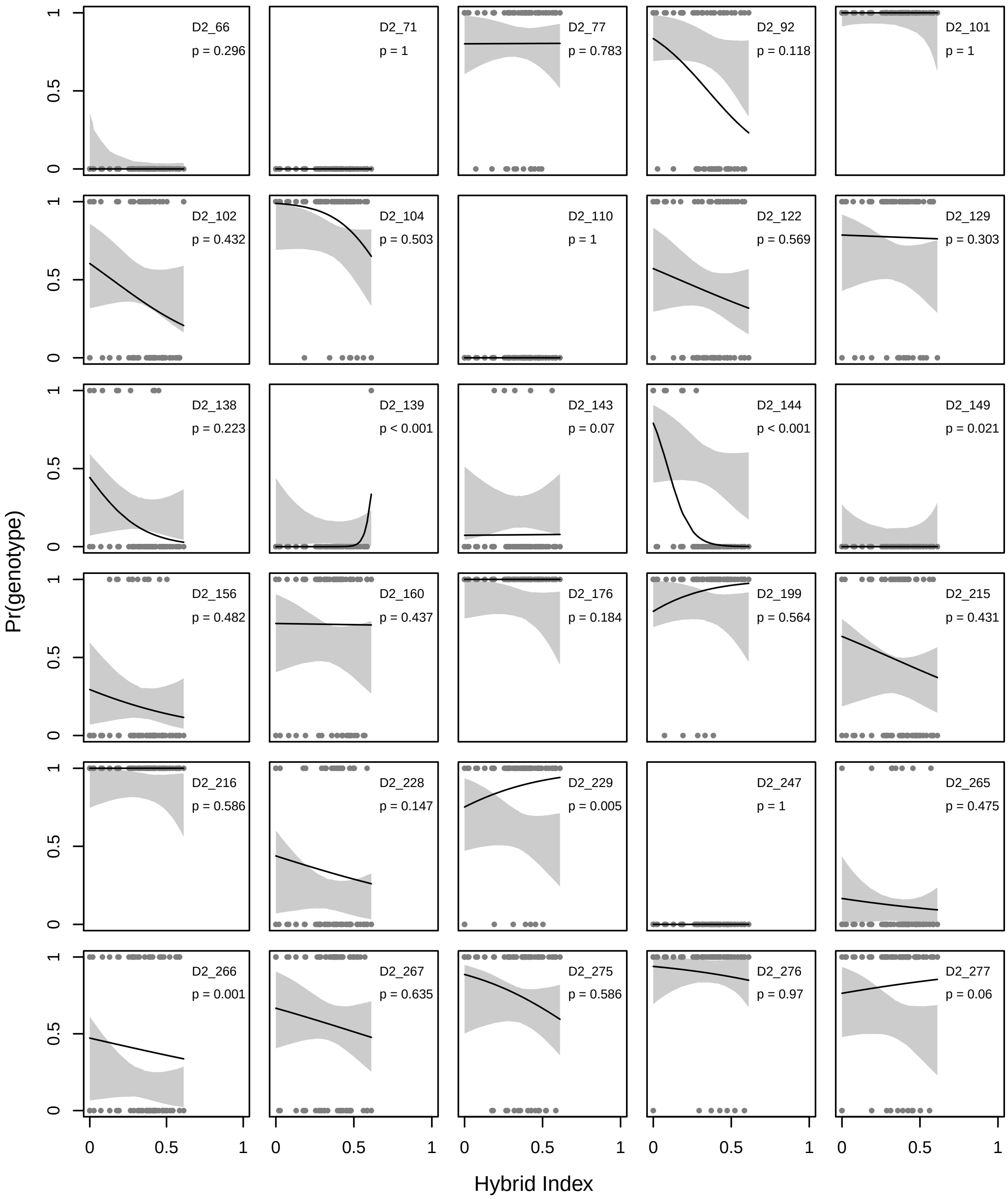

Supp_Figure 2a. Genomic clines for the combined dataset of hyb-1 and hyb-2, as estimated with the R package `introgress`. (grey area) Confidence interval for neutral clines, (lines) actual clines observed. (points top and bottom) Dominant genotype observed in individuals of the two hybrid populations. A hybrid index of 0 indicates a genomic composition as *R. aganniphum*, and an index of 1 as *R. phaeochrysum*.

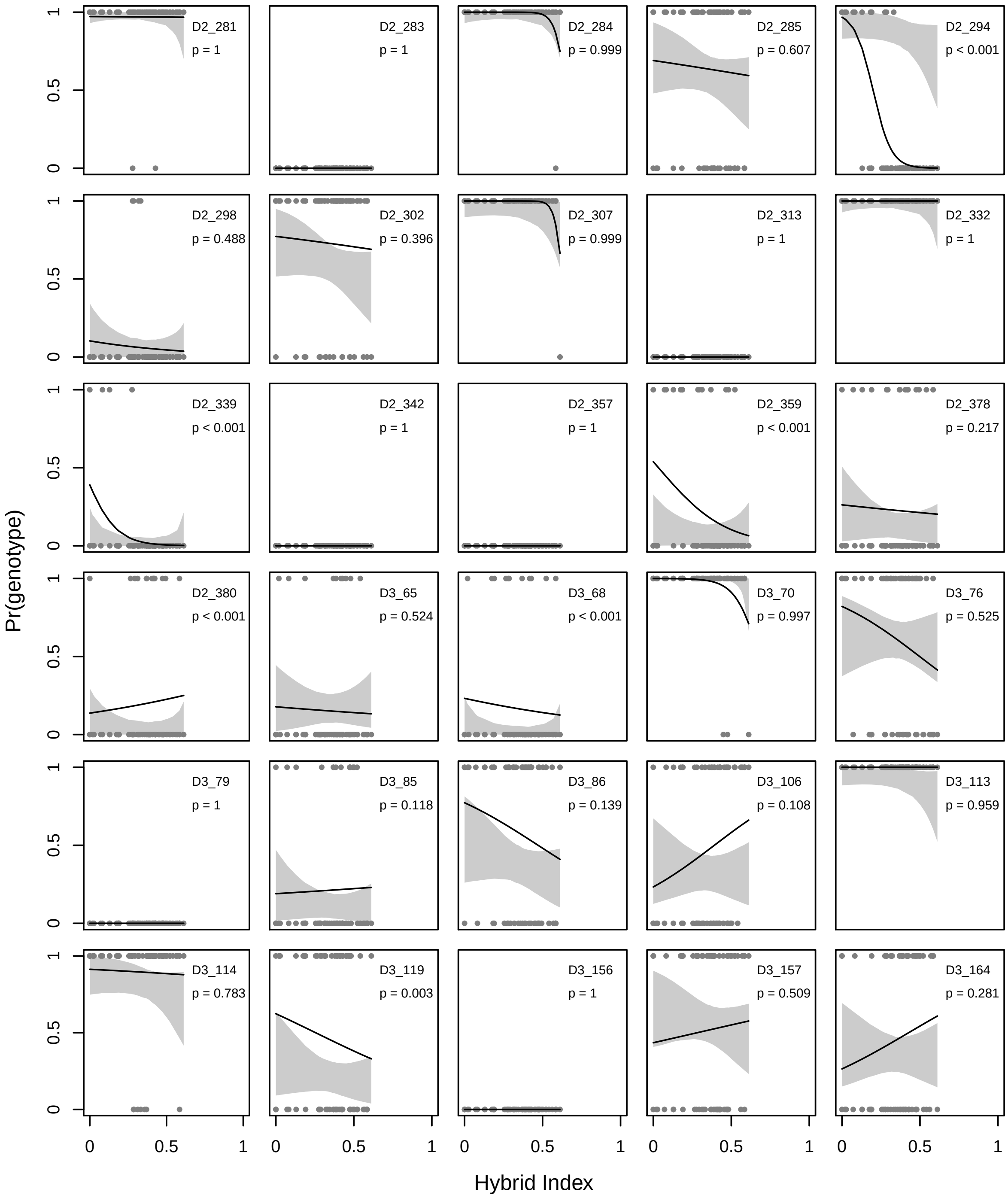

Supp_Figure 2b. Genomic clines for the combined dataset of hyb-1 and hyb-2, as estimated with the R package `introgress`. (grey area) Confidence interval for neutral clines, (lines) actual clines observed. (points top and bottom) Dominant genotype observed in individuals of the two hybrid populations. A hybrid index of 0 indicates a genomic composition as *R. aganniphum*, and an index of 1 as *R. phaeochrysum*.

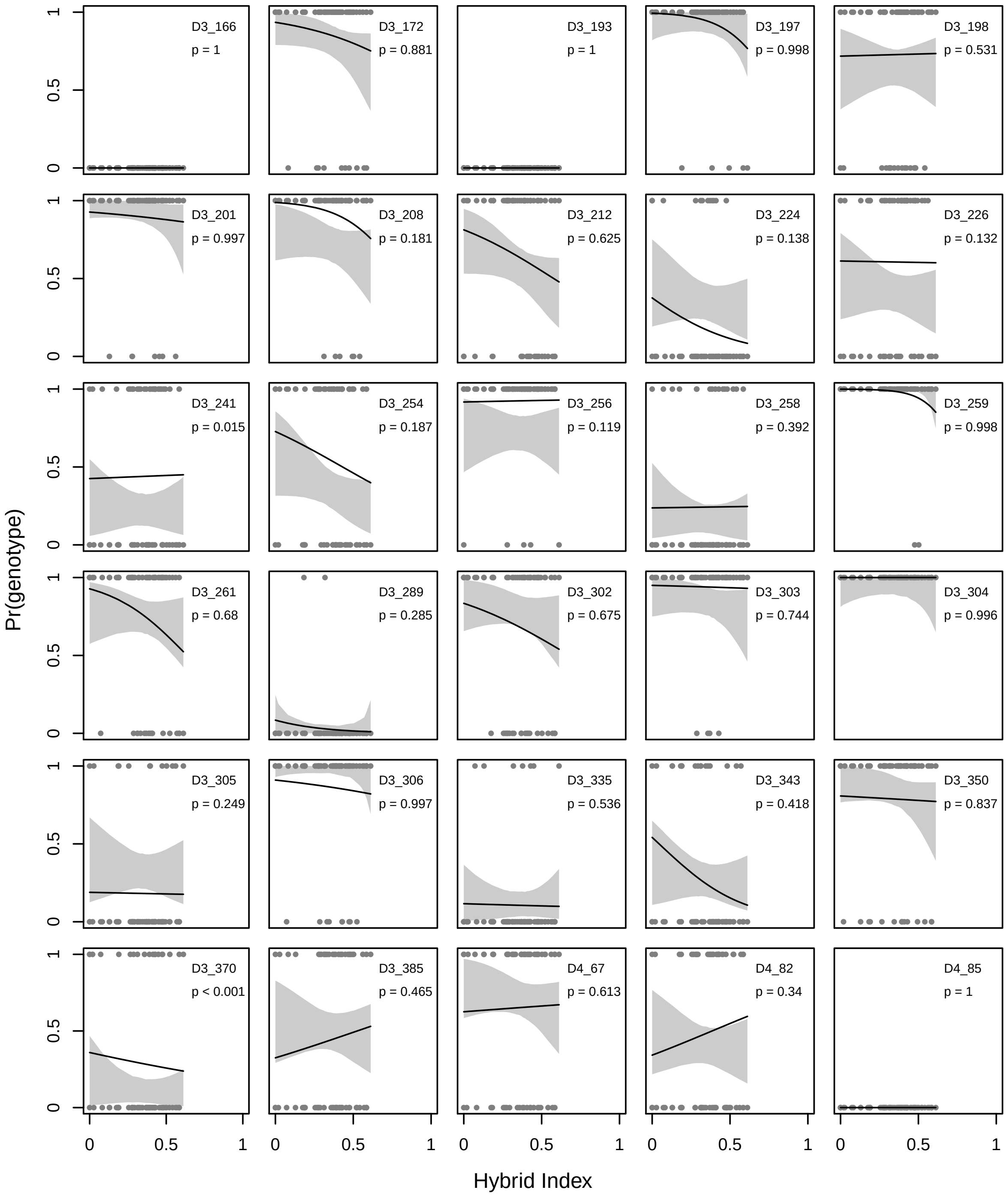

Supp_Figure 2c. Genomic clines for the combined dataset of hyb-1 and hyb-2, as estimated with the R package `introgress`. (grey area) Confidence interval for neutral clines, (lines) actual clines observed. (points top and bottom) Dominant genotype observed in individuals of the two hybrid populations. A hybrid index of 0 indicates a genomic composition as *R. aganniphum*, and an index of 1 as *R. phaeochrysum*.

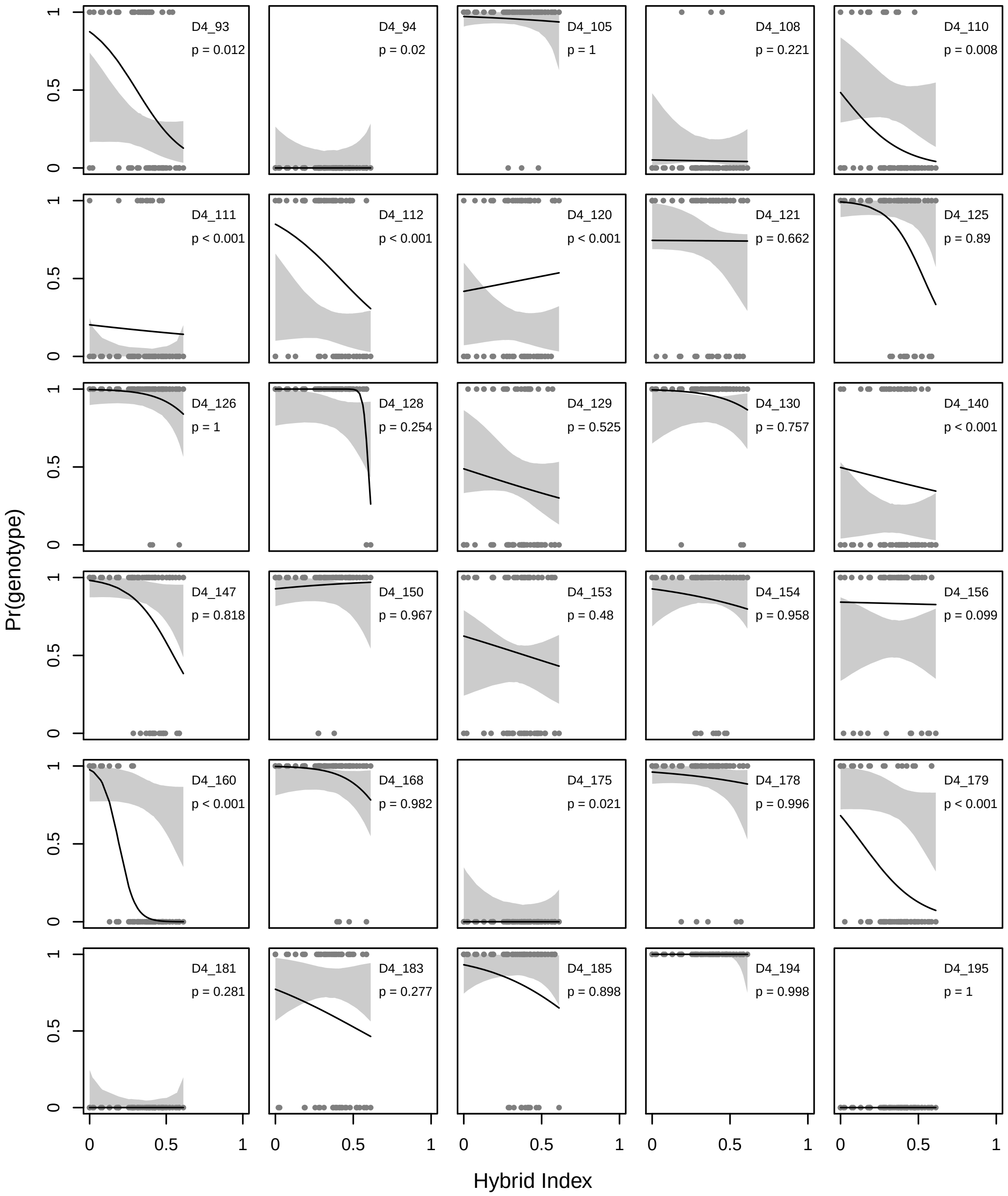

Supp_Figure 2d. Genomic clines for the combined dataset of hyb-1 and hyb-2, as estimated with the R package `introgress`. (grey area) Confidence interval for neutral clines, (lines) actual clines observed. (points top and bottom) Dominant genotype observed in individuals of the two hybrid populations. A hybrid index of 0 indicates a genomic composition as *R. aganniphum*, and an index of 1 as *R. phaeochrysum*.

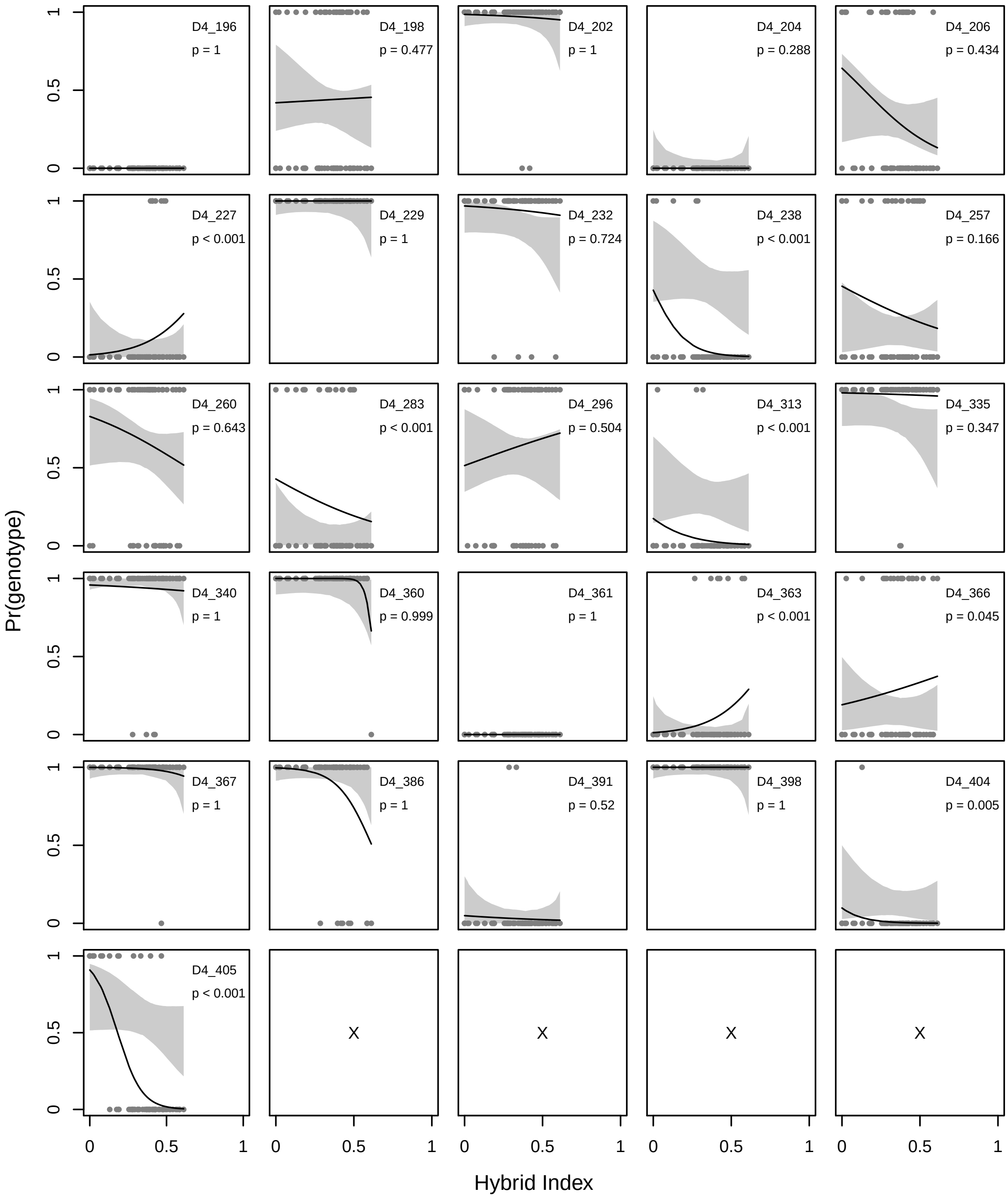

Supp_Figure 2e. Genomic clines for the combined dataset of hyb-1 and hyb-2, as estimated with the R package `introgress`. (grey area) Confidence interval for neutral clines, (lines) actual clines observed. (points top and bottom) Dominant genotype observed in individuals of the two hybrid populations. A hybrid index of 0 indicates a genomic composition as *R. aganniphum*, and an index of 1 as *R. phaeochrysum*.

Only hybrid population hyb-1

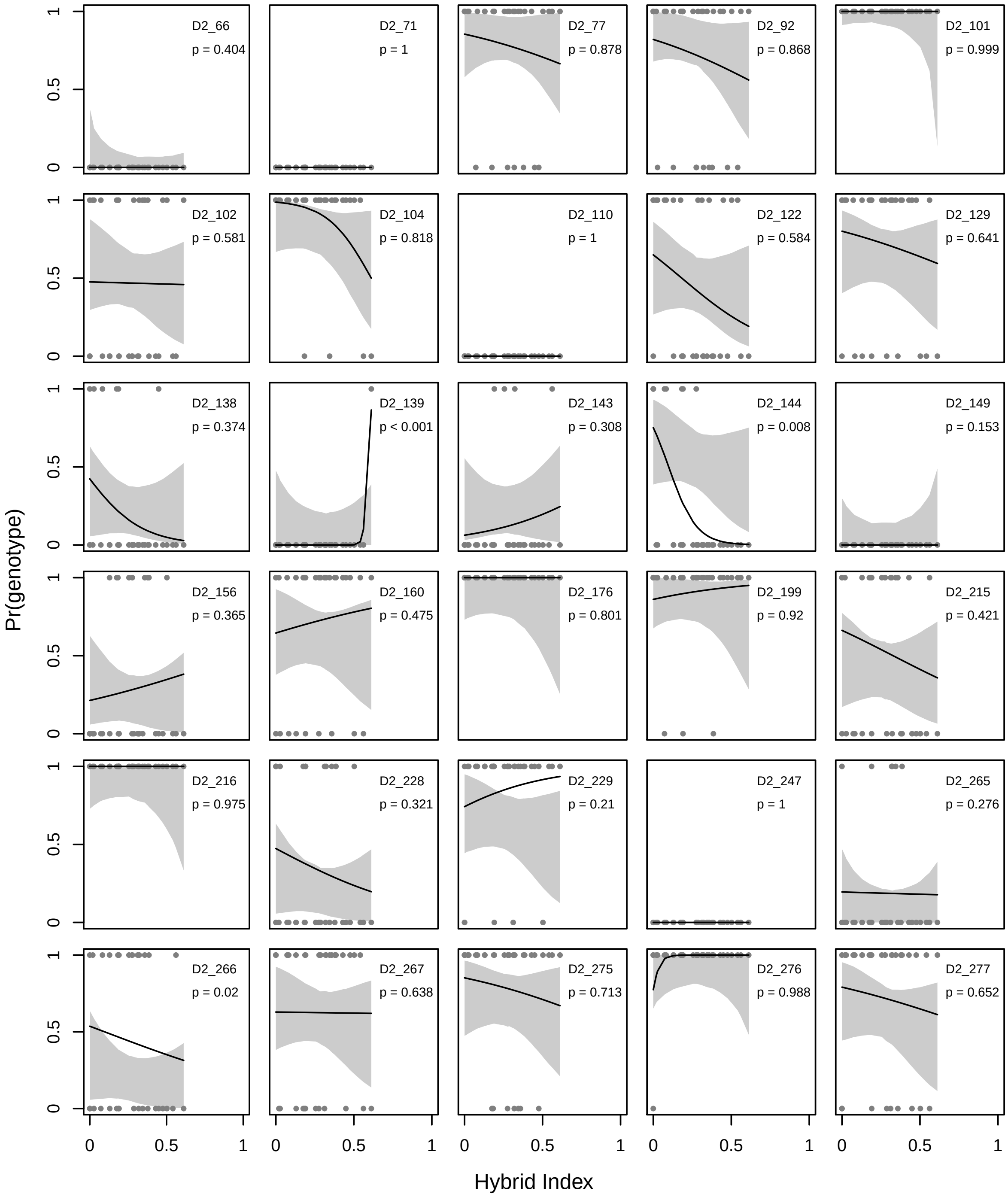

Supp_Figure 3a. Genomic clines for the population hyb-1, as estimated with the R package `introgress`. (grey area) Confidence interval for neutral clines, (lines) actual clines observed. (points top and bottom) Dominant genotype observed in individuals of the two hybrid populations. A hybrid index of 0 indicates a genomic composition as *R. aganniphum*, and an index of 1 as *R. phaeochrysum*.

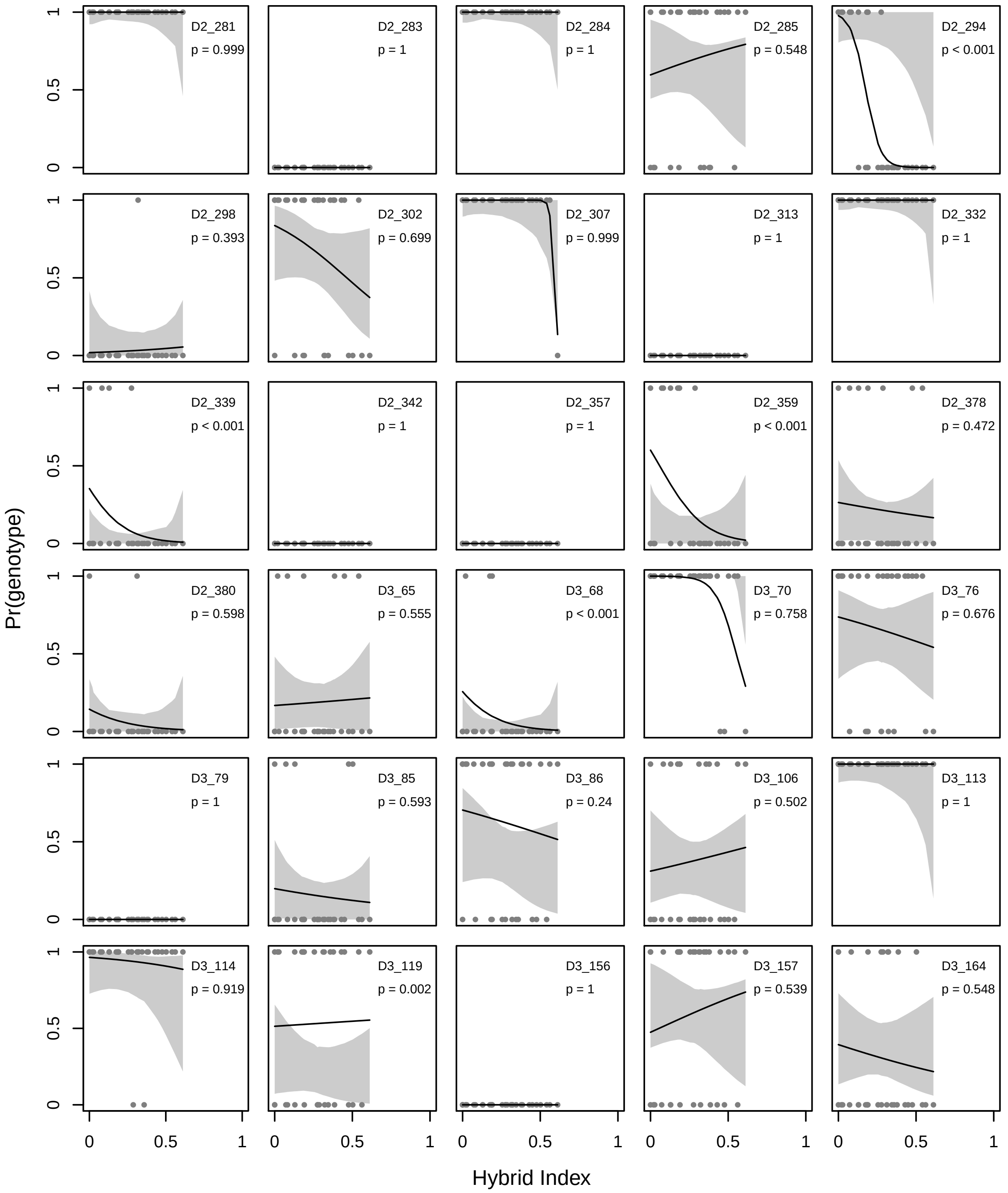

Supp_Figure 3b. Genomic clines for the population hyb-1, as estimated with the R package `introgress`. (grey area) Confidence interval for neutral clines, (lines) actual clines observed. (points top and bottom) Dominant genotype observed in individuals of the two hybrid populations. A hybrid index of 0 indicates a genomic composition as *R. aganniphum*, and an index of 1 as *R. phaeochrysum*.

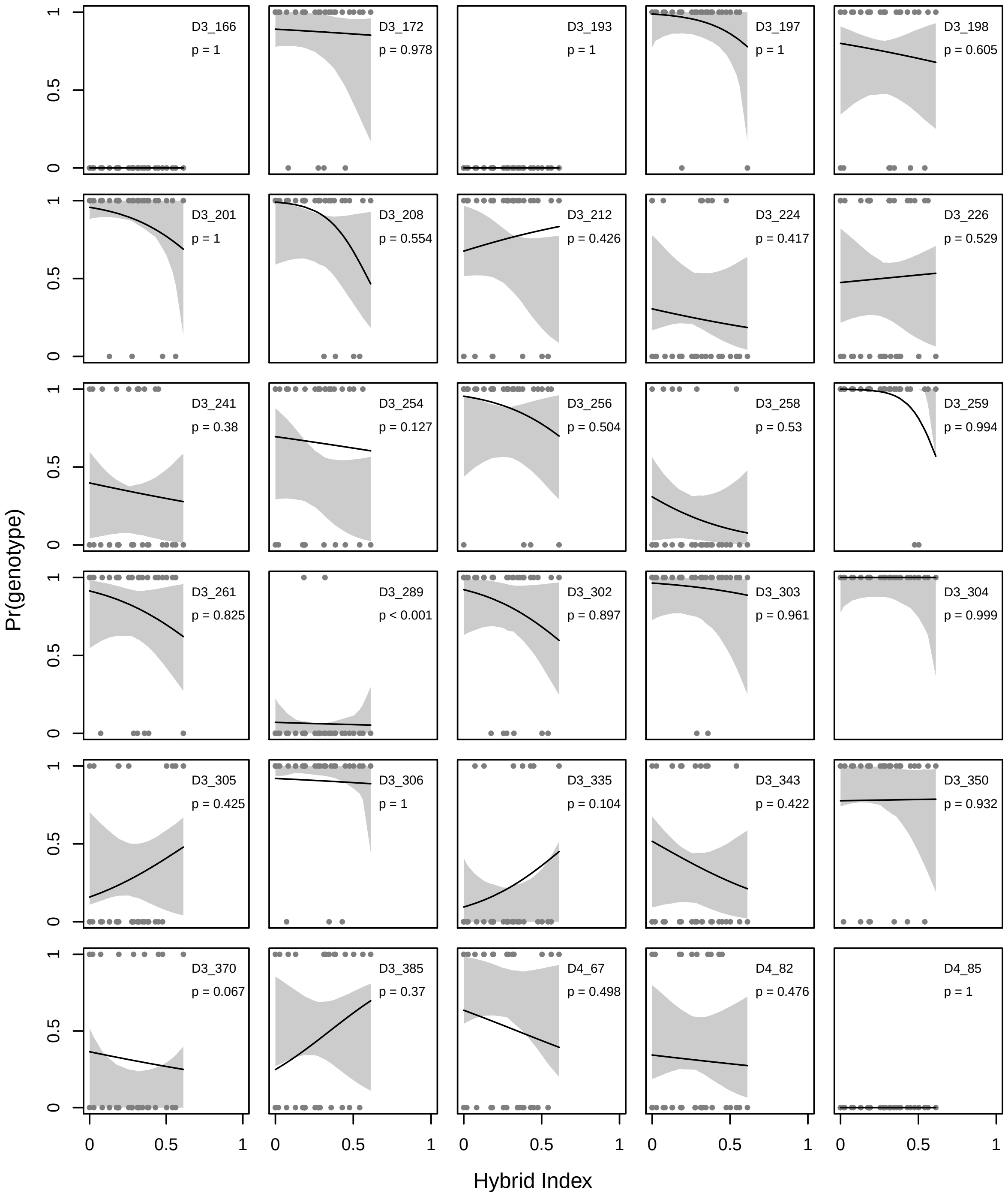

Supp_Figure 3c. Genomic clines for the population hyb-1, as estimated with the R package `introgress`. (grey area) Confidence interval for neutral clines, (lines) actual clines observed. (points top and bottom) Dominant genotype observed in individuals of the two hybrid populations. A hybrid index of 0 indicates a genomic composition as *R. aganniphum*, and an index of 1 as *R. phaeochrysum*.

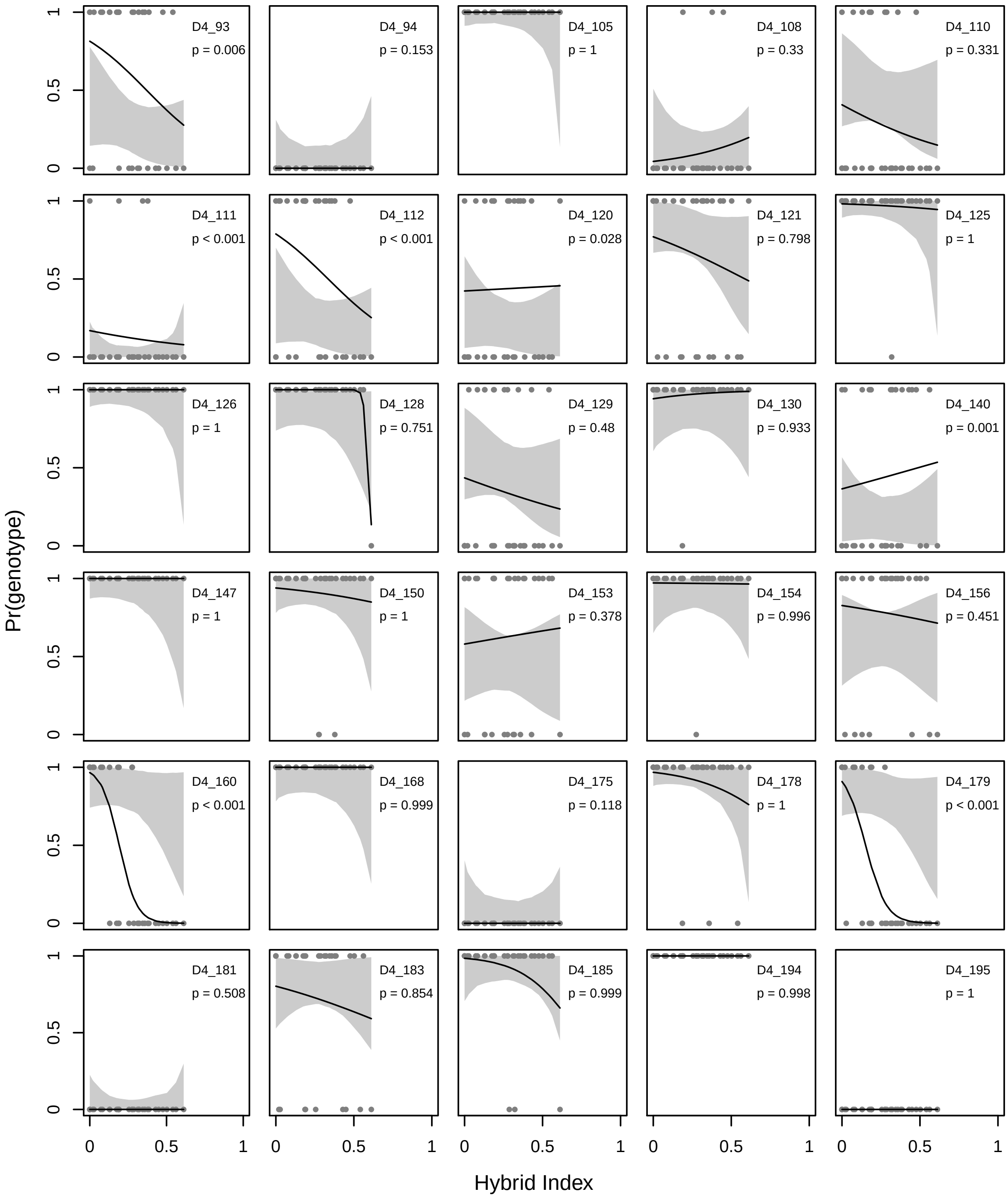

Supp_Figure 3d. Genomic clines for the population hyb-1, as estimated with the R package `introgress`. (grey area) Confidence interval for neutral clines, (lines) actual clines observed. (points top and bottom) Dominant genotype observed in individuals of the two hybrid populations. A hybrid index of 0 indicates a genomic composition as *R. aganniphum*, and an index of 1 as *R. phaeochrysum*.

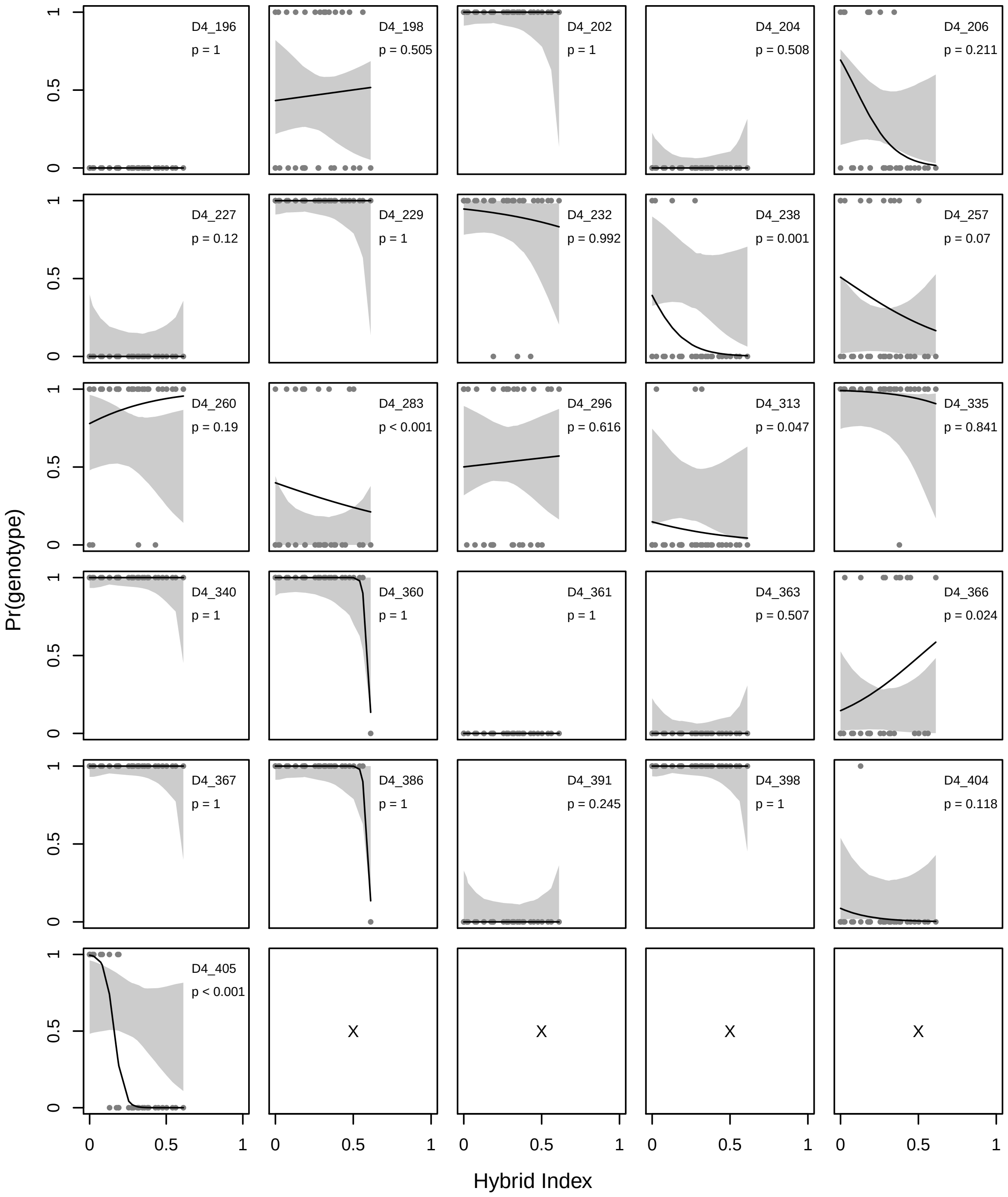

Supp_Figure 3e. Genomic clines for the population hyb-1, as estimated with the R package `introgress`. (grey area) Confidence interval for neutral clines, (lines) actual clines observed. (points top and bottom) Dominant genotype observed in individuals of the two hybrid populations. A hybrid index of 0 indicates a genomic composition as *R. aganniphum*, and an index of 1 as *R. phaeochrysum*.

Introgress: hybrid index for hybrid simulations

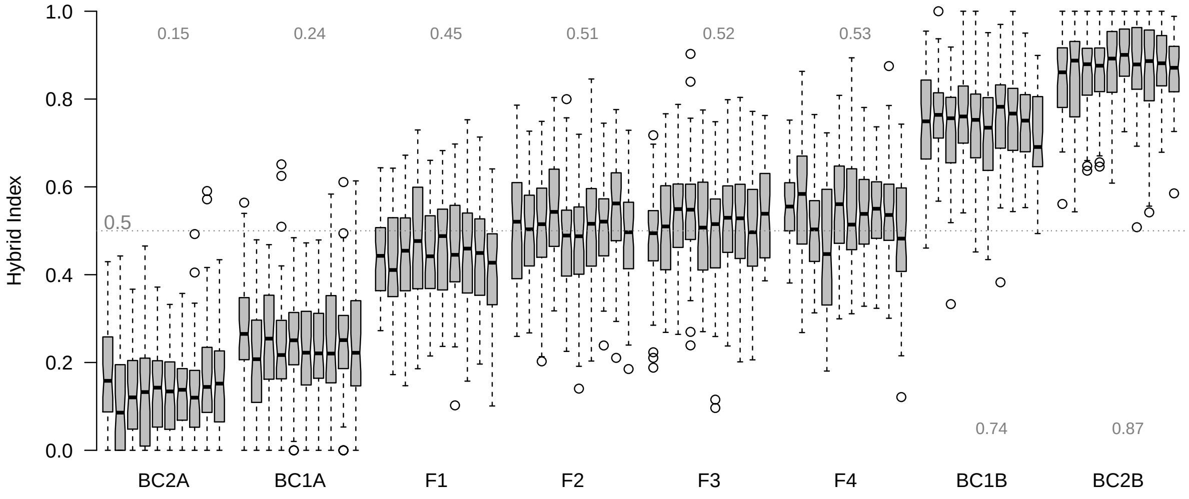

Supp_Figure 4. Mean hybrid indices as calculated by the R package `introgress` for 80 simulated hybrid populations. Each of the simulated populations comprised 50 individuals, and ten populations were simulated for each of eight hybrid classes (backcrosses to *R. aganniphum* (A) – BC2A, BC1A; backcrosses to *R. phaeochrysum* (B) – BC2B, BC1B; F1; inbred later-generation hybrids – F2-F4). Always ten hybrid populations that represent the same type of hybrid class are grouped together; the mean for each group is indicated as grey number above (BC2A to F4) or below (BC1B and BC2B) the bars.

Pairwise F_{ST} histograms

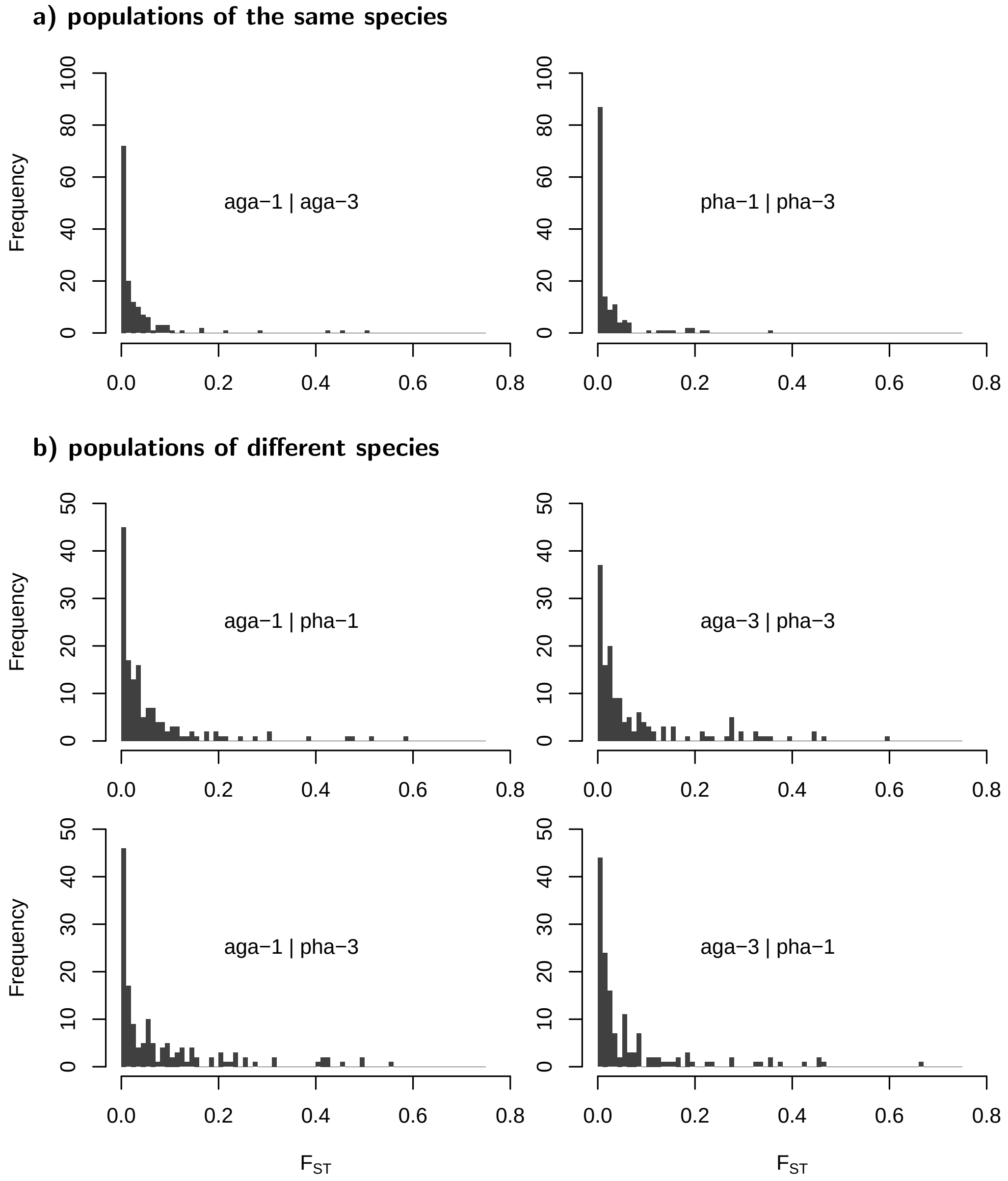

Supp_Figure 5. Histograms of observed pairwise F_{ST} values between populations.

References

Evanno G, Regnaut S, Goudet J (2005) Detecting the number of clusters of individuals using the software STRUCTURE: A simulation study. *Molecular Ecology*, **14**, 2611–2620.
